# Development and application of a quantitative bioassay to evaluate maize silk resistance to corn earworm herbivory among progenies derived from Peruvian landrace Piura

**DOI:** 10.1371/journal.pone.0215414

**Published:** 2019-04-16

**Authors:** Miriam D. Lopez, Tesia S. Dennison, Tina M. Paque, Marna D. Yandeau-Nelson, Craig A. Abel, Nick Lauter

**Affiliations:** 1 Corn Insects and Crop Genetics Research Unit, United States Department of Agriculture, Ames, Iowa, United States of America; 2 Department of Plant Pathology and Microbiology, Iowa State University, Ames, Iowa, United States of America; 3 Genetics and Genomics Graduate Program, Iowa State University, Ames, Iowa, United States of America; 4 Department of Entomology, Iowa State University, Ames, Iowa, United States of America; 5 Department of Genetics, Development and Cell Biology, Iowa State University, Ames, Iowa, United States of America; Ecole des Mines d'Ales, FRANCE

## Abstract

Corn earworm (CEW), *Helicoverpa zea* (Boddie), (Lepidoptera: Noctuidae), is a major insect pest of corn (*Zea mays* spp. *mays* L.). CEW larvae feed on silks, kernels and cobs, causing substantial yield and quality losses both through herbivory and by vectoring pathogens. The long-term goal of this work is to elucidate the genetic and biochemical basis of a potentially novel CEW resistance source discovered in silk tissue of Piura 208, a Peruvian landrace of maize (PI 503849). We developed a quantitative CEW bioassay and tested it on four populations that contrast alleles from Piura 208 with those from GT119, a CEW-susceptible maize inbred line. In replicated analyses of two populations of F_1:2_ families, corn genotype accounts for 84% and 68% of the variance in CEW larval weights, and up to 60% of the variance in CEW pupation percentage, demonstrating both the success of the quantitative bioassay and the strength of the Piura 208 resistance mechanism. Analyses of two corresponding populations of BC_1:2_ families revealed substantially diminished effects of corn genotype on CEW weight gain and pupation. This loss of Piura 208-derived CEW resistance during backcrossing suggests complex (multi-genic) inheritance of a threshold-dependent mechanism. Technical factors in bioassay performance were also assessed, often by analyzing the 1,641 CEW larvae that were raised on control diet (meridic with no corn silks added). Minor, but statistically significant impacts on CEW weight gain, pupation, and mortality were attributable to multiple technical factors in the preparation, incubation and evaluation phases of the bioassay, demonstrating the importance of randomization, stratification, replication, and variable-tracking across the many steps of this quantitative CEW bioassay. Overall, these findings indicate that this scaled-up, quantitative CEW bioassay is fundamentally sound and that Piura 208-derived resistance alleles are experimentally tractable for genetic and mechanistic research using this approach.

## Introduction

Corn earworm, *Helicoverpa zea* (Boddie), (Lepidoptera: Noctuidae), is a widespread and well-studied insect pest of corn [1–5]. The larval stage of the corn earworm (CEW) is responsible for damage to many additional crops, including cotton, tobacco, beans, tomato, soybean, pepper, and many more, although maize is the preferred host. Adult CEWs are capable of long-distance migration; for example, populations in Iowa migrate from Southern states during each growing season. As a multivoltine pest with a short developmental period, facultative diapause, and high female fecundity, CEWs cycle through several generations even within a single temperate growing season, allowing for annual population expansion to damaging levels following initial colonization [1,2]. Female moths oviposit on emerged maize silks where the larvae begin feeding, eventually tunneling down the silk channel to feed on kernel and cob tissues. Larval feeding leads to yield and grain quality losses through pollination interference, vectoring of bacterial and fungal pathogens, and herbivory. Maize silks vary in suitability as a food source for CEW development, with some varieties facilitating fast growth and complete development, and others causing larval growth retardation and even death in some cases [3]. Preventing or slowing down CEW growth and development can provide significant benefits to corn producers, particularly if the exponential phase of CEW local population expansions can be reduced or eliminated.

Currently, control of CEW on maize is largely achieved by planting transgenic varieties expressing insecticidal crystal proteins [4–7], by planting varieties with native resistance properties [8], or by application of pesticides [9]. Pesticide applications for control of CEW must be carefully timed to reach larvae before they tunnel down the silk channel, which requires frequent applications to achieve control [10]. Alternative sources of insect resistance are of interest as a substitute or complement for these control strategies, especially given that insect populations have developed or have the potential to develop resistance to several transgenic control measures [11–14]. One widely known native resistance mechanism is maysin, a naturally-occurring compound found in maize silks that inhibits CEW larval growth [15–19]. Maysin has been extensively studied and alleles favoring maysin production have been incorporated into elite germplasm for control of CEW [20].

A potentially novel form of genetic resistance to lepidopteran pests has been identified and partially characterized in Piura, a Peruvian landrace of maize. Landrace Piura did not experience the same breeding and selection pressures as maize developed in the U.S. Corn Belt, and therefore may harbor beneficial alleles not present in U.S. germplasm. Piura 208 (PI 503849 seed accession) has been shown to decrease herbivory by larvae of multiple lepidopteran pests while exhibiting very low levels of known plant-derived resistance metabolites, including dihydroxy-methoxy-benzoxazinone (DIMBOA), maysin, apimaysin, and 3’methoxy-maysin [3,21,22]. Abel and colleagues [3] identified entry 107-8-7 as a valuable CEW-resistant second generation backcross (BC_2_) accession that arose from a cross between Piura 208 and maize inbred B94. Entry 107-8-7 showed equivalently high levels of CEW resistance in silk bioassays as compared to Zapalote Chico, a highly resistant experimental check; both caused ~12-fold reduction in larval weights at eight days after eclosion and ~20-day delays in time to pupation compared to CEWs raised on diet that included silks from the susceptible check [3]. Notably, 107-8-7 silks had 6-fold lower maysin and 8-fold lower apimaysin+3’methoxymaysin content compared to Zapalote Chico silks, yet showed equivalent resistance [3], suggesting that metabolites other than maysin contributed to resistance to CEW.

Investigation of genetically diverse maize lines may reveal novel native resistance factors, but ascertaining the genetic basis and inheritance of resistance factors requires the ability to test segregating genetic populations in a replicated study. Field tests often measure plant responses such as yield, or a rating of CEW damage, or even the number of insects found per plant. However, because CEW larvae are cannibalistic and territorial, the most reliable information is obtained when only one CEW is present per maize ear, which is difficult to achieve for thousands of experimental plants per study. Moreover, measuring direct impacts of corn genotypes on CEWs in a field experiment is impractical because microclimate and microhabitat are too variable, and because artificial infestation of CEW neonates at a field scale limits data quality if conditions are not optimal.

To measure the effects of silk feeding while minimizing unwanted sources of variation, we have expanded both the scale and scope of a dried-silk bioassay method [3,23] that uses meridic diet amended with freeze-dried, powderized maize silks from experimental as well as known CEW-susceptible and -resistant genotypes. This highly replicated bioassay method allows for the assessment of 1,024 CEW larvae per run, where a run allows data collection on the effects of up to 40 silk samples over 11 days of CEW larvae feeding. To permit analysis of procedural and environmental variables, microclimate data were collected and technical variables were tracked throughout the preparation, incubation, and evaluation phases of the bioassay runs. The bioassay also includes high numbers of control larvae that are fed on meridic diet without silks, allowing technical variation to be analyzed in the absence of diet composition effects.

For this study, we derived four analysis populations that can interrogate the contrast between alleles from GT119, a highly susceptible maize inbred line, with those from accession 107-8-7, the (Piura 208 x B94)BC_2_ with the strongest resistance to silk feeding by CEW larvae. Here we report biological results concerning the CEW bioassay performance of diets containing silks collected from six check genotypes and 144 experimental families belonging to the four populations evaluated. We also report technical results that both establish the success and inform the improvement of the quantitative bioassay described herein.

## Materials and methods

### Experimental plant germplasm

To begin breeding for this study, (Piura 208 x B94)BC_2_S_2_ families were derived from seed accession 107-8-7 [3] through two generations of self-pollinations and single-seed descent without further biochemical or entomological selection on the S_1_ generation (S1 Fig). Twenty (Piura 208 x B94)BC_2_S_2_ families were then tested for CEW resistance using a small scale CEW bioassay as described by Guo and colleagues [17]. The two families showing the highest level of CEW resistance were SCE34-6D91001 and SCE34-6D91007, hereafter abbreviated 91001 and 91007 (S1 Fig). From each of these two CEW-resistant sources, pollen from 10 plants was crossed onto silks of GT119, a maize inbred that produces virtually no maysin and is highly susceptible to silk feeding by CEW larvae. Balanced bulks were made from the resulting sets of (GT119 x 91001)F_1_ and (GT119 x 91007)F_1_ ears (S1 Fig). Notably, the inclusion of 10 donor plants per balanced bulk allows more than one allele per locus to be contributed by 91001 and 91007, the respective donor families (S1 Fig). To produce BC_1_ germplasm for the contrasts between GT119 and either 91001 or 91007, 15 F_1_ plants were grown and used as pollen sources for backcrossing with GT119 as the maternal recurrent parent. Balanced bulks were made from the resulting sets of (GT119 x 91001)BC_1_ and (GT119 x 91007)BC_1_ ears (S1 Fig). This breeding method permits comparative CEW resistance evaluation of F_1_ and BC_1_ generations, but does not retain any specific relationships among the individual F_1_ and BC_1_ plants. Because silks from many plants are needed for the quantitative CEW bioassay used in this study, the F_1_ and BC_1_ individual plants that variably inherited Piura 208 alleles were grown and selfed to produce F_1:2_ and BC_1:2_ analysis families for both resistance sources (S1 Fig). Thus, the four analysis populations represent two generations for each of two closely related resistance sources.

### Design of field experiments

All plots for the experiments were planted on May 10^th^, 2012 at the Iowa State University Agricultural Engineering/Agronomy Research Farm in Boone County, Iowa. One hundred forty-four experimental entries were grown and sampled in triplicate, with 36 entries comprising each of the four populations described above (*i*.*e*. F_1:2_ or BC_1:2_ families of sources 91001 and 91007). These four populations were each grown in three randomized complete blocks, with all 12 blocks containing three plots occupied by check entries. Checks included a known CEW resistant inbred line (SC102), a known CEW susceptible inbred line (GT119), and maize inbred lines B73, B94, B97, and Mo17 for their common usage in prior breeding efforts (Respective seed accessions: Ames 10273, PI 511318, PI 550473, PI 539872, PI 564682, and PI 558532). Each of the six check entries was represented six times overall, with stratified assignment to balance them with respect to contrasts between the two sources (91001 versus 91007) as well as between the two population types (F_1:2_ versus BC_1:2_).

Collectively, there were 468 plots in the experiment as follows: 4 populations x 3 replicates x (36 entry plots + 3 check plots). Fifty seeds were planted into each plot, where plots consisted of two 5 m–long rows grown side-by-side (twin-rows). The 936 rows were manually thinned to assure that no two plants were separated by less than 5 cm, resulting in an average stand count per plot of 41.8 plants and an average within-row spacing of 25 cm. Spacing between rows was 75 cm, and alleys of 75 cm were used to separate ranges of rows. The 39 twin-row plots for each experimental block were grown in three ranges of 26 rows each, making the blocks approximately square (17 m x 19 m). The six blocks arising from the 91001 and 91007 resistance source were kept together and the replicates were stratified to make two adjacent grids of 2 blocks by 3 blocks. To reduce edge effects, the entire grid of 12 blocks was surrounded by a border planting of B73, which has a similar mean stature compared to plants belonging to the 144 experimental entries. The experimental design descriptors and data for all variables recorded on a per plot basis are provided (S1 File).

### Silk sampling and processing

Silk emergence from the encasing husk leaves was recorded for each plot and the female flowering time measure, days-to-silk (DTS) was quantified as the number of days between planting and silk emergence for at least 50% of the plants in a plot (i.e. the mid-silk cohort). Silks were collected from the mid-silk cohort three days after silk emergence on a per plot basis, with collections occurring on 13 consecutive days between July 19^th^ and July 31^st^, 2012. To provide sufficient dry silk mass for the CEW bioassay diet, emerged silks were bulk harvested from a minimum of 30 plants per plot into aluminum foil packages and flash frozen in liquid nitrogen within 5 minutes of collection. The frozen foil packages were transported in coolers of dry ice and stored at -80°C. Prior to lyophilization, several small holes were made in each foil package to facilitate rapid, consistent drying of silks. Each 18.9 L vacuum jar held three foil packages that were lyophilized for a minimum of 72 hours at 40 to 50 microbars of vapor pressure in a freeze dryer (Model 77555, Labconco, Kansas City, MO). Dry silk samples were ground to a fine powder using a knife mill (GlenMills Inc., Clifton, NJ). For each sample, an aliquot of 3–6 g of powder was weighed, and stored at -80°C for use as a bioassay diet ingredient.

### CEW bioassay

#### Bioassay design

Although preparation, incubation, and evaluation for each bioassay run spans 12 days (not including CEW moth rearing for egg production), it is designed to commence once per week and distributes the work required across periods of five consecutive days, interspersed by two-day periods with no work requirement. For example, we prepared diets on Wednesdays, infested on Thursdays, and weighed CEWs 11 days post-infestation on Mondays. In this configuration, a one-day delay in hatch for the CEW eggs results in Friday infestations and collection of weight data on Tuesdays, which does not perturb the overall schedule. The capacity of the bioassay was also scaled such that all steps could be performed with 40 or fewer person-hours of effort per week.

Because CEWs are territorial and often cannibalistic, each CEW must be reared in its own cell to ensure that final CEW weights are not influenced by competition or cannibalism. Each bioassay run uses 1,024 CEWs raised in 32 polystyrene trays that have 32 cells each (product #RT32W, Frontier Scientific, Newark, DE). Each silk powder sample originating from a single field plot is mixed with the ingredients of a meridic diet (described below) to create an experimental CEW diet, hereafter called a test diet. Each test diet is fed to 24 CEWs that are distributed in cohorts of 8 into single quadrants in each of three trays, mitigating potential negative effects of infestation order, spatial variation during incubation, and weighing order.

Up to 40 test diets can be evaluated per bioassay run while maintaining at least 6.6% of CEW larvae dedicated to control diet, which does not contain silks. For each bioassay run, test diets were assigned to a batch (4 batches per run) and assigned a diet number within batch (10 diets per batch). Test diets were then prepared in that order, with the purpose of accounting for any differences in larval weight due to timing of diet preparation.

For these experiments, each run evaluated 39 test diets derived from the silk samples originating in a single randomized complete block. This allowed for a minimum of 8.6% of the cells to contain control diet. If a field plot failed to yield enough dry silk powder to prepare a test diet, the entry was replaced in the bioassay run with control diet. This practice ensured that all 12 runs of the bioassay contained diet in all quadrants of the bioassay trays. The three replicates of each population were evaluated in bioassay runs as follows: (GT119 x 91007)F_1:2_ families in February, 2013; (GT119 x 91007)BC_1:2_ families in March, 2013; (GT119 x 91001)BC_1:2_ families in January, 2014; and (GT119 x 91001)F_1:2_ families in April, 2014.

#### Bioassay preparation

Test diets were prepared from each silk sample on a per gram of silk powder basis, as follows: 42.1 ml of water was microwave-heated with 0.93 g of agar until the agar was fully melted. This base was then cooled to ≤ 60°C so that the nutritive diet ingredients would not be chemically altered by heat. Powderized silks were mixed with a commercially-available meridic diet mix (product #F9240B, Frontier Agricultural Sciences, Newark, DE) at a rate of 7.1 g of diet mix per gram of silk powder; this mixture was added to the agar/water base and mixed in a blender. While still liquid, silk tissue diets were dispensed into 3 quadrants (24 cells) distributed across bioassay trays, and allowed to solidify at room temperature for ~2 hours before being stored at 4°C.

To streamline the workload of filling the 128 tray-quadrants (1,024 cells) with up to 40 test diets each week, we implemented a repeatable spatial assignment structure that simplified dispensing diet into tray-quadrants, loading infested trays into the incubation chamber, and maintaining their relative positions during the shift that occurs as the trays for the next run enter the chamber. Notably, the structure preserved the randomizations of experimental entries, ensured even spatial distribution across diet batches, and spatially stratified the three quadrants of eight cells for each diet. Hereafter, we refer to this experimental design feature as “cohort stratification”.

Prior to infestation, diet-filled bioassay trays were removed from the 4°C refrigerator to allow the diets to warm to room temperature. Infestation was performed manually using *H*. *zea* larvae obtained from a laboratory culture maintained by Monsanto Company, St. Louis, MO. *H*. *zea* eggs were shipped in an insulated container with temperature stabilizers appropriate for the season. Once received, eggs were incubated at ~27°C and ~65% relative humidity until hatch. Upon eclosion, a single neonate larva was placed into a cell containing diet using a dampened, fine camel hair brush, and cells were covered with self-adhesive porous lids (product #RTCV4, Frontier Agricultural Sciences, Newark, DE). The investigator performing infestation was tracked for each bioassay tray to permit analysis as a technical factor. The experimental design descriptors and data for all technical and biological variables recorded on a per CEW basis are provided (S2 File).

#### Bioassay incubation

Larval feeding took place over an 11 day period at ~27°C and ~65% relative humidity in an incubation chamber with interior dimensions of 3 m wide x 3 m deep x 2.25 m tall. Temperature was maintained using a Dayton heater and a Witt condenser/evaporator coil for cooling and air circulation. Humidity was maintained using a Lennox Humidispray. A wire-rack shelf system to hold two sets of 32 bioassay trays was located in the center of the room to allow for maximum airflow on all sides, and to prevent an edge effect due to proximity to a wall. The shelf unit is 150 cm wide, 46 cm deep, and 191 cm tall, with 11 shelves occupying the medial 125 cm and spaced 12.5 cm apart. The 32 trays of a run are placed on five shelves in a 6-7-6-7-6 trays-per-shelf configuration. The 11^th^ shelf is needed because the uppermost bioassay trays always have a shelf of empty trays above them so that all trays are exposed to an equivalent amount of overhead airspace (10 cm) throughout the incubation period.

Positional coordinates were tracked for each cell of the bioassay to take into account both vertical and horizontal position on shelves, including proximity to shelf edge in both directions. Shelf number is a relative vertical position coordinate, because after 7 days, trays were moved from the top 5 shelves to the bottom 5 shelves to accommodate the trays of the subsequent bioassay run. The positional coordinates were tracked (S2 File) to permit investigation of weight or developmental differences influenced by microclimate (distance from heat source, lights, humidity gradients, edge effects, etc.). Microclimates inside the incubator were monitored at the top, middle, and bottom on the left and right sides of the shelf unit using sensors for temperature and relative humidity (HOBO part # U12-012, Onset Computer Corporation, Bourne, MA). The semi-continuous microclimate data at six positions in the incubation chamber for the duration of all 12 bioassay runs is provided (S3 File).

#### Bioassay evaluation

After 11 days of incubation, the 1,024 CEWs are removed from their cells and individually weighed using an analytical balance (Practum 224-1S, Sartorius AG, Goettingen, Germany), with care taken to remove diet and frass prior to weighing. Three additional binary outcomes were recorded, including pupation, death prior to measurable weight gain, and death during incubation after measurable weight gain. The mortality designations are hereafter termed “dead at infestation” and “died after growing”. Because experimental diets of this type are not known to be sufficiently toxic to cause immediate death, we treat this third class as missing data, since it is likely that the neonate was lost or accidentally killed during infestation. As such, this binary measure is only evaluated as a technical factor for bioassay preparation.

### Statistical analyses

The impacts of biological and technical factors on CEW weight gain were assessed using analysis of variance (ANOVA) or generalized linear models, with application of the Bonferroni multiple-test correction to identify significant terms [24]. Whenever multiple terms were simultaneously fit, inclusion of each term in the results required significance at α = 0.05 and that the term improved the adjusted R^2^ value as compared with each less complex model. Comparisons of means following ANOVAs were conducted using the Tukey honestly significant difference (HSD) or Tukey-Kramer HSD tests, depending on whether or not the sample sizes used to calculate the means were equivalent. All statistical analyses were conducted using JMP Pro 12.0.1 (SAS Institute Inc., Cary, NC).

## Results and discussion

Measuring inhibition effects of corn silks on CEW herbivory in a laboratory bioassay presents several challenges of scale and scope. To assure agricultural relevance of results, a robust genetically diverse colony of CEWs must be used, necessitating that experimental diets each be tested using at least a moderate-sized cohort of CEWs. In turn, this requires both a large amount of silk tissue per entry and a large incubation space. As each phase of the effort scales up, achieving consistency or being able to account for unavoidable inconsistencies increases the scope of the effort. To assess 439 silk-based test diets in this study, ~20,000 corn plants were raised to maturity and monitored daily during flowering to control the duration of silk exposure to the environment, which is known to affect the surface lipid metabolome of silks [25,26] that has been implicated in CEW resistance in prior studies [27–29]. Silks that had emerged from encasing husk leaves were specifically collected because CEW moths oviposit on emerged silks and thus this is the tissue CEW neonates initially feed on. Emerged silks collected from ~14,000 of these plants were then processed into test diets that were fed to ~12,000 CEWs raised in individual cells within an incubation chamber. Genetic differentiation among silk samples was assessed using mean CEW weights from 24 insects per test diet and three independent test diets per genetic entry. Technical aspects of the bioassay were also assessed using metadata collected from all phases of the process. The analysis of technical factors was conducted on a per insect basis, in some cases using only the data from CEWs that were fed control diet. Additional data analyzed in this study include daily weight measurements of CEWs raised on control diet, flowering time (days-to-silk) for the 439 plots, and microclimate measurements at six positions within the incubator across the bioassay runs, all of which are used for interpretation of biological and technical results.

### CEW weight gain is affected by the genetic source of maize silks

**Fig 1:**
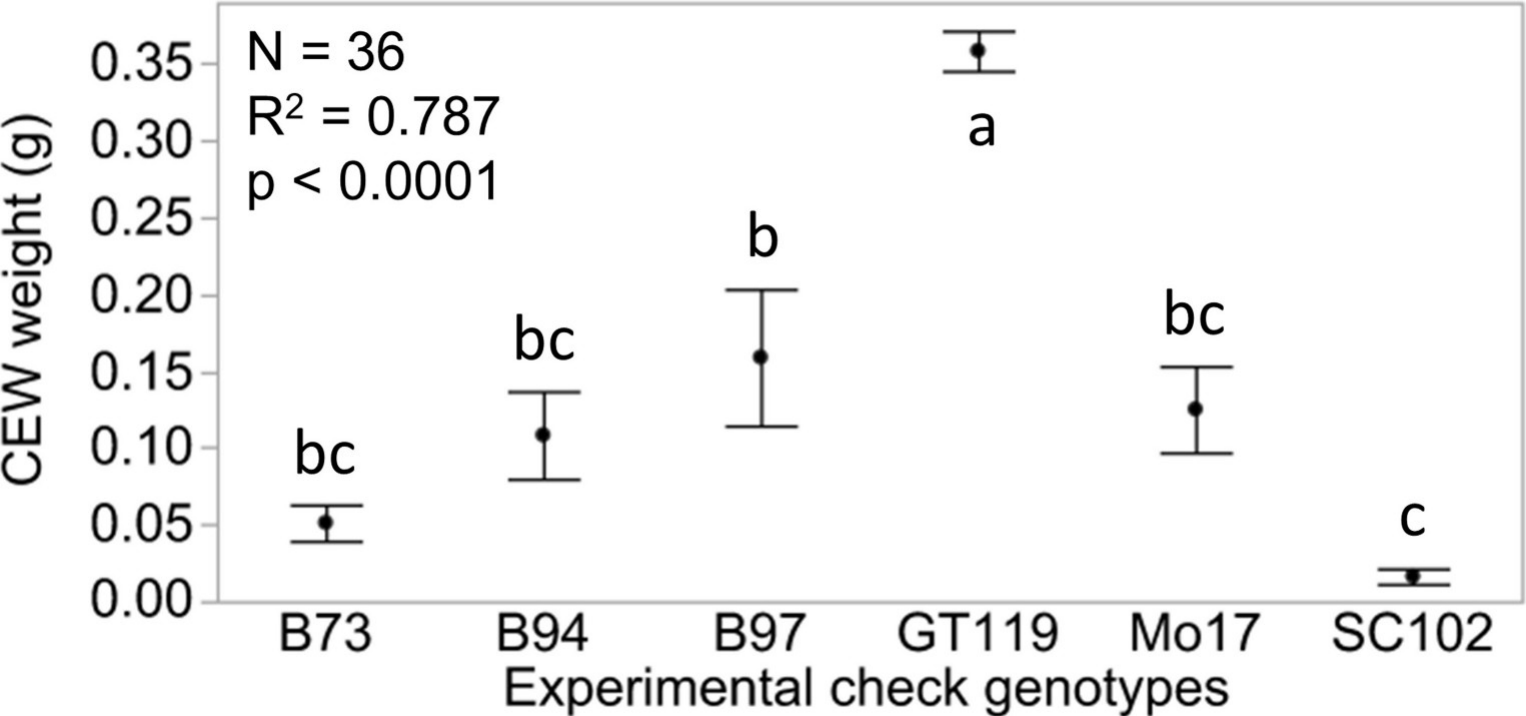
CEW weight gain differentiates silk-based diets produced from resistant and susceptible experimental check genotypes. For each of the six genotypes, six plots were grown and assayed using 24 CEWs each. Averages of these CEW weights generated 36 plot-level data points, which are summarized here as means ± SE for each check genotype. ANOVA summary statistics are provided.

Maize silks are known to vary in suitability as a food source for CEW development [3]. In this bioassay, dried silk tissue replaced 12.3% by weight of the nutritive ingredients of a meridic diet optimized for lepidopteran larvae. We expect that maize silks provide sustenance for CEW larvae due to their commonality as oviposition substrates. However, when comparing dried silk-containing diets with control diet in a bioassay, the net decrease in nutritional value could theoretically be 12.3%, if we presume dried silks provide no nutritional value. If the relative reduction in weight gain of CEWs fed on dried-silk diets vs. control diet is caused by nothing more than decreased density of nutritive ingredients, we would expect all dried silk-based diets to equally impact CEW weight gain, which was not the case. We capitalized on the known resistance of SC102 and susceptibility of GT119 as an initial test of the dried silk CEW bioassay. Across a group of six experimental checks grown in six plots each, maize inbred line accounted for 79% (ANOVA, p<0.0001) of the variance in CEW weight gain, demonstrating that the bioassay can reveal both strong (*e*.*g*. GT119 versus B73) and more subtle (*e*.*g*. B97 versus SC102) differences in the ability of silks from particular inbred lines to limit CEW weight gain in the bioassay (Fig 1).

**Fig 2:**
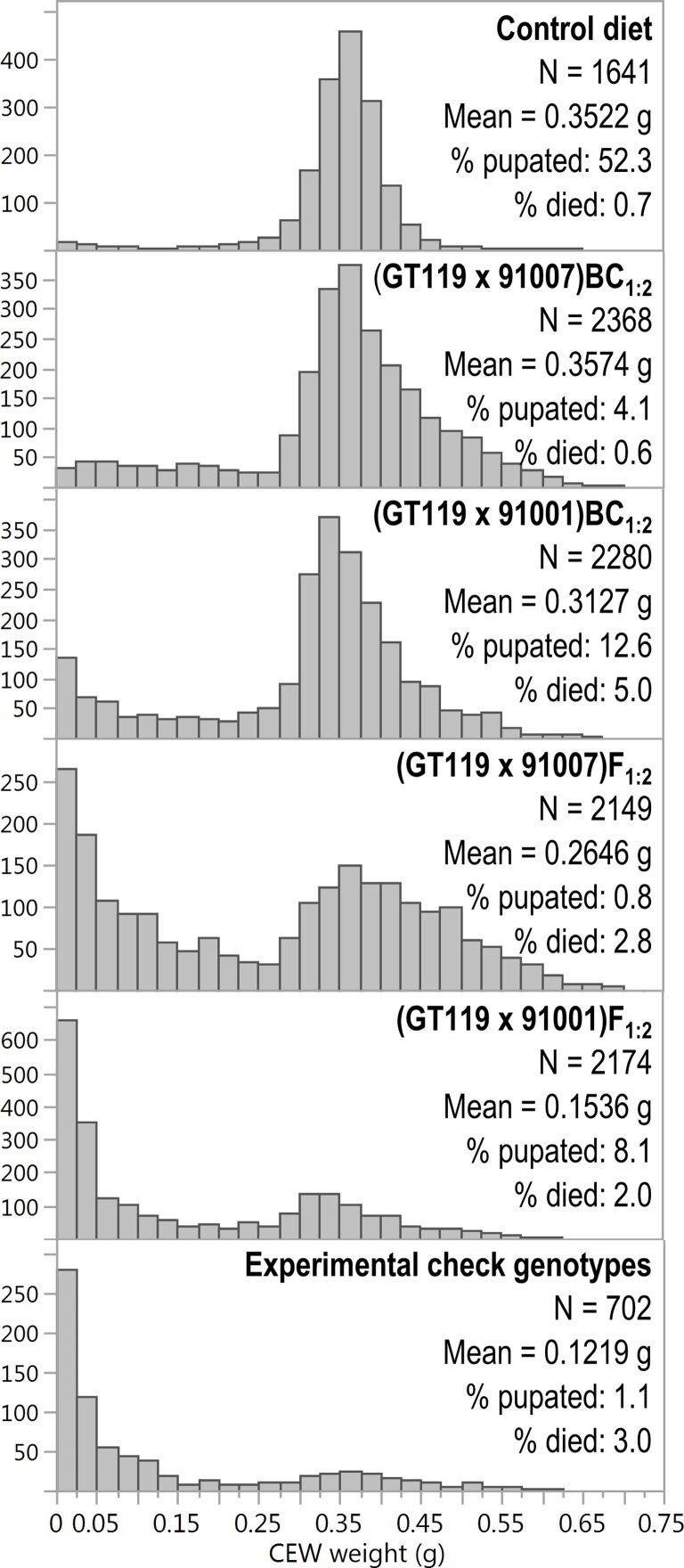
Distributions of CEW weights reveal differences in bioassay performance across control diet, four populations that segregate for resistance alleles, and experimental check genotypes. Histograms depict 11-day post infestation CEW weights. The y-axes (scaled individually for each bioassay group), show counts for each of the uniformly-scaled bins for CEW weight as defined on the x-axis. Sample sizes and sample means are provided. The histograms are ordered from top to bottom by ascending resistance level. Mean weight, percent pupated, and percent died are also reported. In all, weights for 11,314 CEWs are represented, including those from the 266 larvae (2.4%) that initially grew and developed, but died during the incubation period (S2 File).

**Fig 3:**
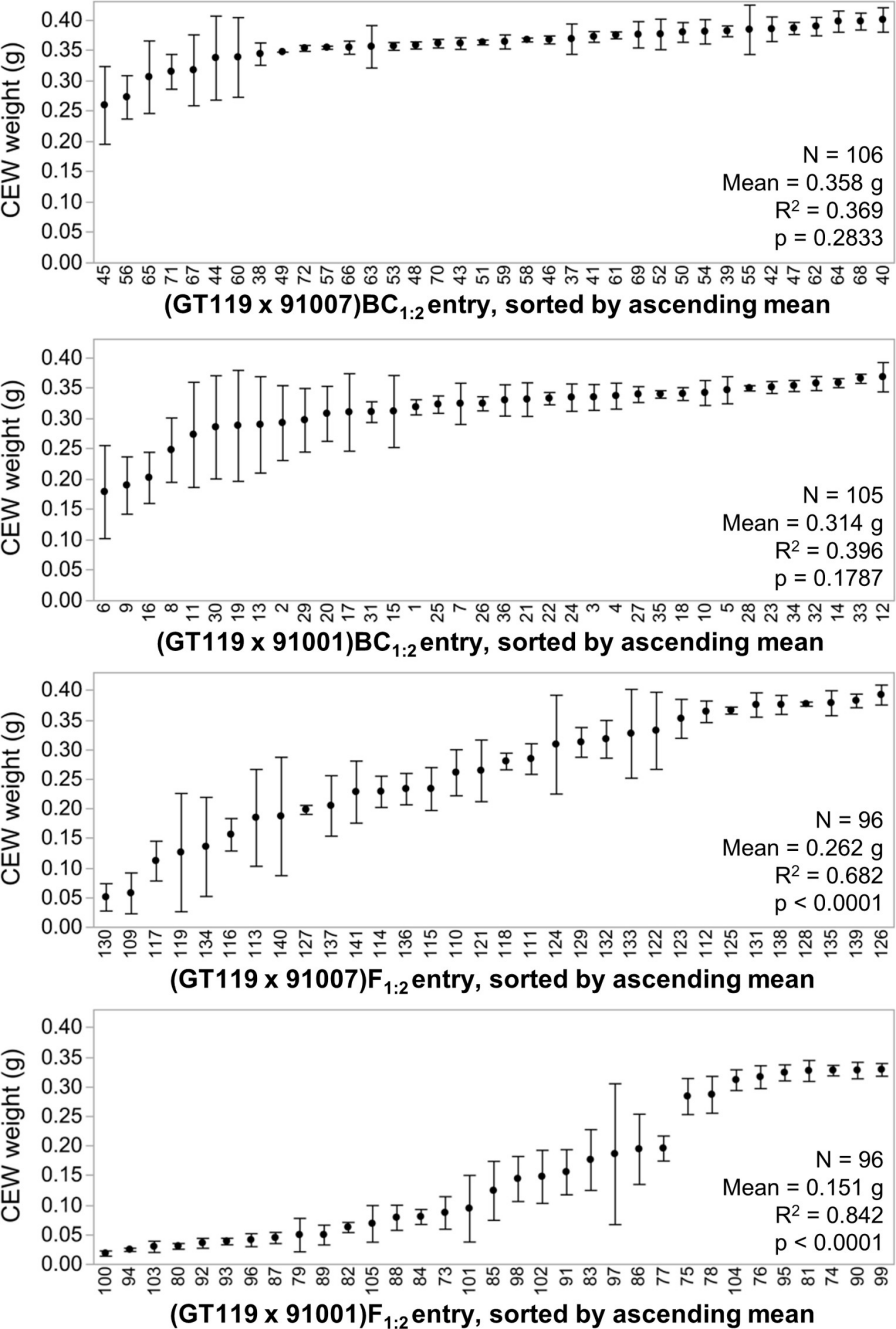
Variation in CEW weight is explained by genetic source of maize silks to varying degrees across four populations that segregate for resistance alleles. CEW weight means are shown for each entry with standard error bars indicating the levels of variation among the sets of test diets. ANOVA summary statistics are reported for each population. Sample sizes are the numbers of test diets assayed for 36, 36, 32, and 33 entries, respectively. For 130 of these 137 entries, data were obtained for all three replicates. For comparison, the four populations appear in the same order as in Fig 2.

The bioassay was also successful in quantitatively differentiating segregating F_1:2_ genetic families. Of the 4 populations tested, silks from (GT119 x 91001)F_1:2_ entries conferred the greatest overall levels of resistance to CEW feeding as evidenced by the low weight gain of larvae tested in the bioassay (Fig 2). While the overall mean weight was 0.154 g across all 2,174 CEWs raised on diets containing silks from 33 entries of the (GT119 x 91001)F_1:2_ population, nearly one third of these CEWs weighed less than 0.025 g (Fig 2). By comparison, larvae tested on control diet had a mean weight of 0.352 g (Fig 2), while larvae tested on CEW resistant SC102 silks had a mean weight of 0.015 g (Fig 1). The differential response observed among the (GT119 x 91001)F_1:2_ entries has a clear genetic basis; 84% (p<0.0001) of the variance in CEW weight gain can be attributed to genetic entry within this population (Fig 3). Entries from the (GT119 x 91007)F_1:2_ population also exhibited strong differential resistance to CEW feeding, with an overall mean CEW weight of 0.265 g (Fig 2) and 68% (p<0.0001) of the weight variation attributable to genetic entry (Fig 3). However, only 10% of these CEWs weighed less than 0.025 g, suggesting that the 91007 resistance source is not as strong as the 91001 source (Fig 2).

The bioassay was unable to quantitatively differentiate segregating BC_1:2_ genetic families in both the 91001- and 91007-derived BC_1:2_ populations, which is indicated by non-significant p-values in the ANOVAs for CEW weight by entry (Fig 3). No BC_1:2_ entries showed levels of resistance comparable to the best F_1:2_ entries in either the 91001 or 91007 background (Fig 3). Moreover, using a Tukey-Kramer HSD test, neither the mean weights of CEWs raised on (GT119 x 91007)BC_1:2_ nor (GT119 x 91001)BC_1:2_ silks were significantly different from mean weights for CEWs raised on control diet (p = 0.9999 and p = 0.8042, respectively; Fig 3). The relative inability of the BC_1:2_ families to prevent CEW weight gain is suggestive of multi-genic inheritance of a threshold-dependent mechanism. Focused genetic dissection efforts will be required to characterize the modes of inheritance and allele actions for the Piura 208 CEW resistance trait.

### CEW weight gain is not associated with silk collection date

Flowering time in all four populations is strongly affected by entry (Table 1 and S1 File), introducing the potential to confound weather and genetic source as explanatory factors affecting CEW weights. Specifically, because silks from all three replicates of each entry were typically collected during a narrow time span within the 13-day collection period, it is possible that variable daily weather conditions could induce changes in the silk metabolome, and by extension, contribute to CEW weight differences. Given that all of the replicates from this study were collected in a single growing season, such a contribution would end up being statistically attributed to entry. To consider whether the strong influence of entry on CEW weight could be attributable to silk growth environment, we tested for associations between flowering time and CEW weight gain within each population, and found none (Table 2). We also addressed this concern by comparing the relative explanatory power of collection date and entry as model terms to account for variance in CEW weights. Using plot-level data from all four populations (N = 403) in a two term linear model, entry has a significant effect (R^2^ = 0.787, p < 0.0001), whereas collection date does not (R^2^ = 0.008, p = 0.3904). Therefore, we conclude that the differences in CEW weight attributed to entry can be primarily explained by genotypic effects, rather than by genotype X environment effects.

**Table 1:** ANOVAs for flowering time by genotypic entry in four populations.

Test Group	N_a_	R^2^	DF_b_	F ratio	Prob >F
**(GT119 x 91007)BC**_**1:2**_	106	0.91	35	19.78	p <0.0001
**(GT119 x 91001)BC**_**1:2**_	105	0.91	35	18.87	p <0.0001
**(GT119 x 91007)F**_**1:2**_	96	0.96	31	55.38	p <0.0001
**(GT119 x 91001)F**_**1:2**_	96	0.87	32	13.55	p <0.0001

_a_ For ANOVAs for the four populations, sample size is the number of field plots measured.

_b_ Degrees of freedom are based on number of corn genotype entries in each ANOVA.

**Table 2:** ANOVAs for CEW weight gain by silk collection date in four populations.

Test Group	N_a_	R^2^	DF_b_	F ratio	Prob >F
**(GT119 x 91007)BC**_**1:2**_	106	0.056	105	0.72	0.6761
**(GT119 x 91001)BC**_**1:2**_	105	0.104	104	1.09	0.3765
**(GT119 x 91007)F**_**1:2**_	96	0.062	95	0.63	0.7656
**(GT119 x 91001)F**_**1:2**_	96	0.090	95	0.84	0.5952

_a_ Sample sizes report the number of field plots observed and corresponding test diets assayed.

_b_ Degrees of freedom are based on number of corn genotype entries in each ANOVA.

### CEW incubation for 11 days is suitable for testing silk-augmented diets

**Fig 4:**
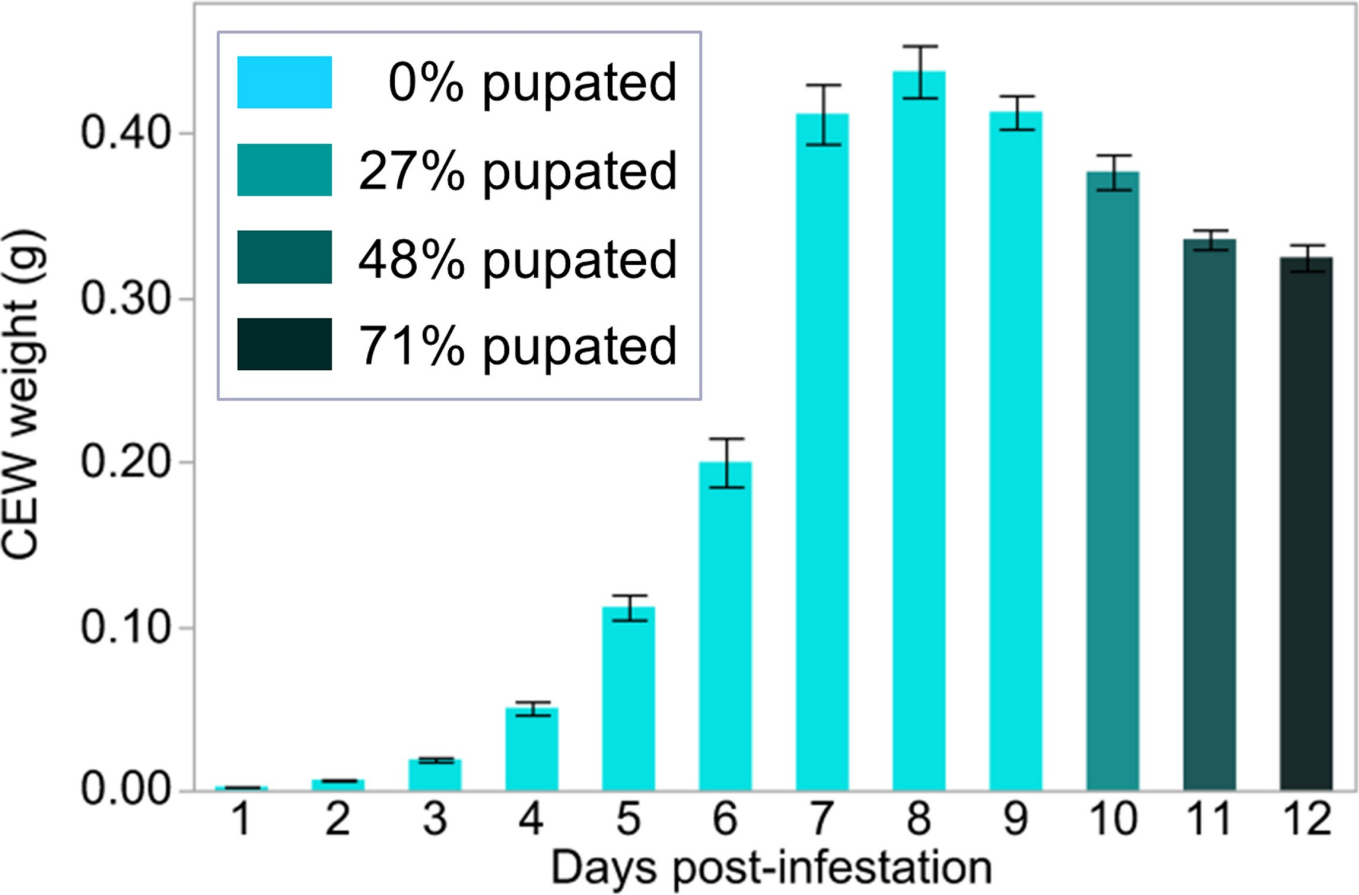
Dynamics of CEW growth and development on control diet demonstrate weight loss associated with pupation. CEW neonates were placed in 576 individual cells of control diet so that a random cohort of 48 CEWs could be weighed on each of 12 days post-infestation. Mean CEW weights ± SEs are reported, with the proportion of pupated CEWs indicated by bar color.

**Fig 5:**
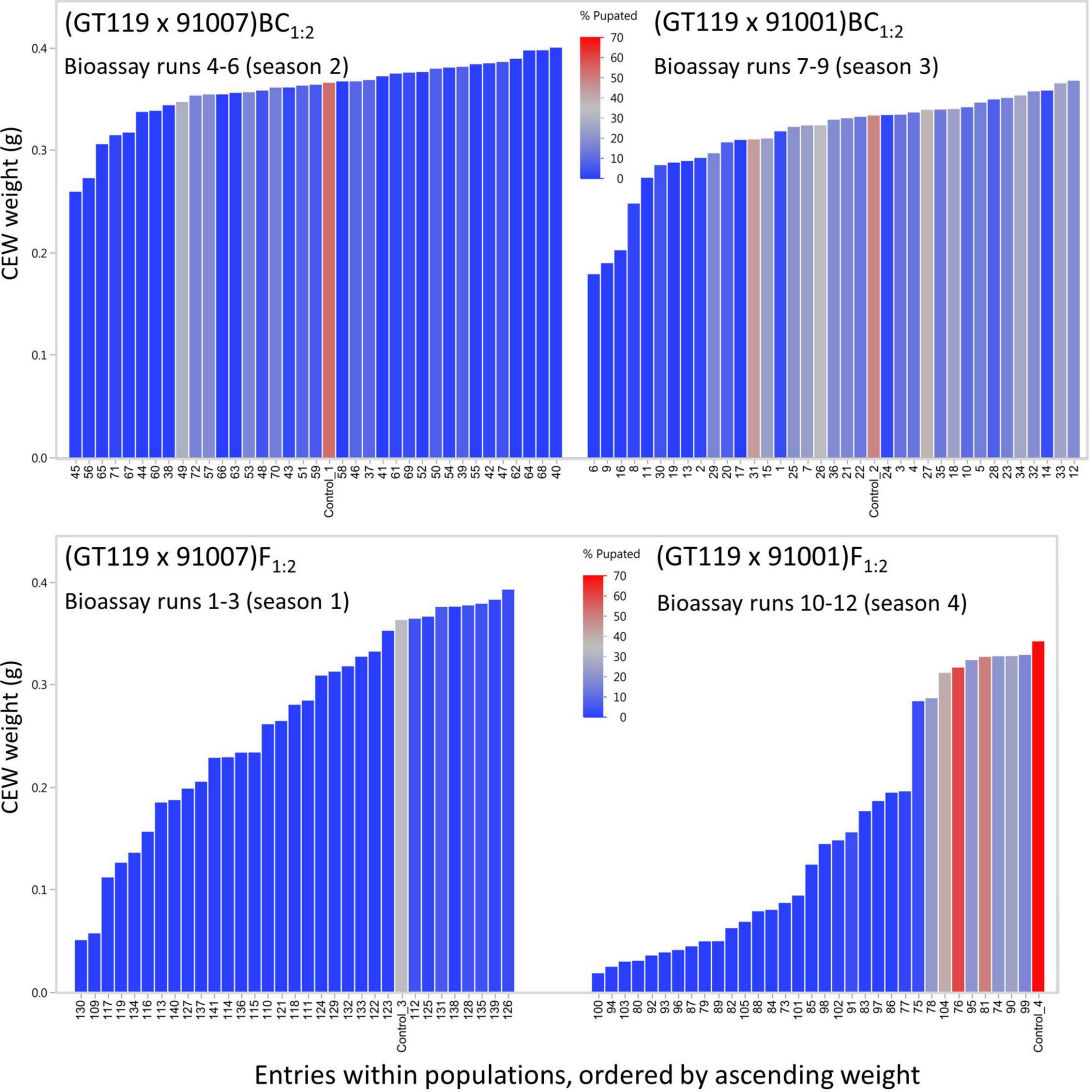
Mean CEW weight and pupation percentage are variable and associated. Entry means obtained from up to 72 CEWs per entry at 11 days of incubation are ordered by ascending CEW weight, allowing visualization of positive associations between weight gain and pupation percentage in several of the populations. Note pupation percentage on control diet varies across the populations much more dramatically than does CEW weight gain, suggesting at least a minor role for technical factors that vary across the temporal scale of 12 bioassay runs.

For a bioassay that quantifies insect weight gain as an end-point measurement, choosing the length of the incubation period is critical and should be based on both logistical and biological considerations. Previous CEW bioassay methods have used an eight day incubation period [23,30,31]. For this study, we vetted an 11-day incubation period to allow for overall increases in throughput and efficiency while maximizing differentiation of CEW responses to test diets (see Materials and Methods). To assess the effects of lengthening the bioassay, we conducted an experiment using 576 CEWs feeding on control diet. Cohorts of 48 CEWs were randomly assigned to be weighed and subsequently discarded each day for 12 days after infestation. Weight gain and pupation data for these 576 CEWs raised on control diet revealed that weight loss is associated with preparing for and undergoing pupation, and that nearly half of the CEWs had pupated by day 11 (Fig 4). However, an 11 day incubation period is not necessarily too long when the purpose is to differentiate responses to silk test diets, because supplementing control diet with corn silks often results in decreased CEW weight gain and/or slower progression to pupation (Figs 2, 3 and 5). Across 12 runs of the bioassay, CEW weights were lower for a majority of insects raised on diets containing silk tissue as compared with those raised on control diet (Figs 2 and 5). Moreover, only 5.6% of 9,673 CEWs raised on diets containing silk tissue pupated by day 11, compared with 52.3% of the 1,641 CEWs raised on control diet (Figs 2 and 5).

### CEW pupation is affected by the genetic source of maize silks

Although only 5.6% of CEWs raised on silk-augmented diets pupated, average pupation percentage across 143 entries ranged from 0% to 61%, suggesting that genetic source affects pupation. Indeed, we observed a statistically significant effect of genotypic entry on pupation percentage for three test groups, with the clearest association in the (GT119 x 91001)F_1:2_ population, in which 60% of the variability in pupation was attributable to entry (Fig 5 and Table 3). A second clear case is seen for the check genotypes (Table 3), for which only CEWs raised on diets containing silks from the susceptible inbred line GT119 pupated (S1 File). In the (GT119 x 91007)BC_1:2_ population, entry affects pupation more weakly, but at a statistically significant level, perhaps because low levels of pupation across most of the entries are juxtaposed with a few entries that have modest pupation percentages averaged across three replicates (Fig 5, Table 3 and S1 File). In the remaining test groups, the conclusion that genotypic entry affects CEW pupation percentages is not strongly contradicted. In one case, pupation is universally low, and in the other, it varies modestly without any stark contrasts (Fig 5 and Table 3). Overall, we conclude that genetic source of silks has a meaningful effect on whether or not CEW larvae developmentally advance to pupae within the 11-day bioassay incubation period. These results are consistent with previous findings, which showed that silk augmentation of meridic bioassay diet can lead to substantial delays in pupation [3,32].

**Table 3:** ANOVAs for percentage of CEWs pupated by genotypic entry.

Test Group	N_a_	R^2^	DF_b_	F ratio	Prob >F
**(GT119 x 91007)BC**_**1:2**_	106	0.47	35	1.75	p = 0.0234
**(GT119 x 91001)BC**_**1:2**_	105	0.41	35	1.40	p = 0.1184
**(GT119 x 91007)F**_**1:2**_	96	0.33	31	1.03	p = 0.4425
**(GT119 x 91001)F**_**1:2**_	96	0.60	32	2.93	p < 0.0001
**Experimental check genotypes**	36	0.33	5	2.95	p = 0.0279

_a_ For ANOVAs for the four populations and the checks, sample size is the number of test diets assayed.

_b_ Degrees of freedom are based on number of corn genotype entries in each ANOVA.

The basis for how silk-based test diets from particular genotypic entries reduce percentage pupation appears to correspond closely with effects on CEW weight gain (Fig 5). This is expected, because a CEW larva must progress through five or six successively larger instars to achieve developmental competence to undergo pupation, and these repeated moltings are each typically accompanied by larval weight gain (Fig 4). To more carefully explore the relationship between CEW weight gain and pupation, we tested for significant associations on a per diet basis. Strong associations were observed between CEW weight gain and pupation percentage in four of five test groups (Table 4). For both GT119 x 91001 populations, the relationship is visually apparent in the data (Fig 5). However, the results are less clear for the two GT119 x 91007 populations. In one case, the (GT119 x 91007)BC_1:2_ population, there was no association between weight and pupation percentage, which could be attributable to insufficient variation in CEW weights across the test diets (Fig 5 and Table 4 and S1 File). For the (GT119 x 91007)F_1:2_ population, the relationship is not visually apparent (Fig 5), but is statistically significant (Table 4). In this case, seven of the 32 entries of this population recorded low, but non-zero pupation percentages and all of them were among the eight highest entries for average CEW weight (S1 File). Such low levels of pupation within a population can create challenges for interpretation. Indeed, for six of the seven (GT119 x 91007)F_1:2_ entries recorded as having pupation, only one of the three replicates recorded a non-zero pupation value, and 75% of these occurred in bioassay run #2 (S1 File). This observation accounts for the failure to statistically detect a relationship between entry and pupation percentage for this population (Table 3), even though the relationship between CEW weight and pupation is evident (Table 4).

**Table 4:** ANOVAs for pupation percentage by CEW weight.

Test Group	N_a_	R^2^	DF_b_	F Ratio	Prob >F
**(GT119 x 91007)BC**_**1:2**_	106	0.001	105	0.09	p = 0.7631
**(GT119 x 91001)BC**_**1:2**_	105	0.054	104	5.85	p = 0.0173
**(GT119 x 91007)F**_**1:2**_	96	0.053	95	5.29	p = 0.0237
**(GT119 x 91001)F**_**1:2**_	96	0.272	95	35.08	p < 0.0001
**Experimental check genotypes**	36	0.217	35	9.43	p < 0.0042

_a_ Sample size represents the number of test diets analyzed.

_b_ Degrees of freedom reflect the number of test diets in each linear model.

### CEW mortality is not attributable to genetic source of maize silks

Rates of CEW mortality during incubation warrant investigation because the inclusion of dried silk tissue in test diets could confer antibiotic properties. Of the 11,314 CEWs that did not die at infestation, 266 (2.35%) were recorded as “died after growing” (S2 File). Death rates by test group ranged from 0.6% to 5.0% (Fig 2), and the distribution of these rates significantly deviated from a Chi-square expectation (p < 0.0001), suggesting that some experimental diets may possess antibiotic properties. To investigate the observed differences across the six bioassay test groups (Fig 2), we examined death rates by bioassay run and found that CEWs died at a disproportionately high rate during bioassay run #9 (S2 File and S2 Fig). Indeed, 108 of the 266 CEWs that died after growing were from run #9, including 93 (11.4%), 9 (10.6%) and 6 (11.3%) larvae that died on (GT119 x 91001)BC_1:2_, control, and experimental check diets, respectively. The other two replicates for each of the 36 (GT119 x 91001)BC_1:2_ entries were tested in bioassay runs #7 and #8, each of which displayed unexceptional levels of CEW mortality, and notably included no deaths occurring on control diet (S2 Fig). Together, these observations indicate that the CEWs used for run #9 were of lower quality than normal. Thus, data from run #9 were excluded from all further analysis of CEW mortality.

We then investigated the relationship between the cohort proportion of CEWs that died after growing (PDAG) and the mean 11-day CEW weight for the living members of the cohort. Across 400 test diets, PDAG accounts for 10.3% of the variance in CEW weight at the cohort level (p < 0.0001). Because these 400 diets are part of 5 different test groups, ANOVAs were performed separately and revealed unequal strengths in this relationship across the four populations and the experimental checks (S1 Table). Notably, the two F_1:2_ populations showed the strongest relationships between PDAG and CEW weight, indicating that the attributes of a test diet that inhibit CEW growth may also contribute to mortality.

To dissect the underpinnings of these differences in mortality, ANOVAs for PDAG by entry were performed and revealed that genetic source of maize silks could not statistically account for variation in mortality in any of the five test groups (S2 Table). The apparent disconnect between the explanatory power of test diet and entry is not irreconcilable; it simply means that the test diets associated with the replicates for each entry did not cause consistent levels of CEW mortality. While this is suggestive of technical inconsistencies, no particular pre-assay process or condition (S2 File) could effectively explain the PDAG variation associated with test diet. In summary, we conclude that the corn silk genotypes tested in these experiments had no discernable impact on CEW mortality during the bioassay. Thus, any potential antibiotic metabolites derived from the silks appear to be non-lethal in this bioassay wherein silk powder comprises only 12.3% of the dry ingredients in the diet.

### Analysis of insect-level data reveals importance of technical factors

Because new challenges associated with the procedural and spatiotemporal aspects were anticipated in this scaled-up CEW bioassay, we tracked a large set of technical factors that could be used to assess their relative importance (S2 File). For tests of these technical factors, appropriate analysis frameworks were chosen such that some investigations utilize only data from the 1,641 CEWs raised on control diet, while others use larger data sets in more complex models that simultaneously account for biological factors such as genetic entry of the silk source. It is important to be aware that the proportion of variance explained drops substantially when analyzing the insect level data as compared with plot-level data. This occurs because much of the variation in weight and pupation among CEWs is due to their individual performance and therefore can not be accounted for at the individual insect level. For example, across 439 test diets, entry (N = 143) explains 82.0% of the variance in CEW weight. However, entry explains only 45.6% of the variance in CEW weight when the same data are used at the insect level (N = 9,673), revealing a gap in explanatory power that must be kept in mind when interpreting the effects of technical factors at the insect level.

#### Variability introduced by investigators

Two cases were observed wherein non-biological variance is statistically attributable to the investigator who performed the work. In one case, CEW mortality at infestation was significantly higher for Individual 3 than for the other investigators performing neonate infestations (S3 Fig). Individual 3 infested just 1.8% of the neonates, but 21.5% of the CEWs that were ‘dead at infestation’ were infested by Individual 3 (S2 File). In the second case, the individual recording CEW weights had a minor, yet statistically significant impact on final CEW weights (R^2^ = 0.021, p < .0001) (S4 Fig). This effect could be an artifact of timing of weighing; for example, an individual who weighed CEWs later in the day would be weighing insects that had additional time to feed as compared with insects weighed at the beginning of the day. Alternatively, this effect could be due to the degree to which investigators remove meridic diet and/or frass from the CEWs prior to weighing. Either way, CEW weight differences attributable to the individual performing weighing were minor, and were far surpassed by genetic effects of test diets. These results suggest that cohort stratification is an important experimental design feature for minimizing the detrimental effects of technical issues of these sorts. They also illustrate that dexterity and attention to detail are required to successfully perform these fine-motor, repetitive tasks.

#### Variability introduced along spatial gradients during incubation

Because microclimatic variation within any large incubator is expected to exist along spatial gradients, tests for whether the growth cell location during incubation impacted CEW weight and pupation were conducted. Observable, but minor spatial trends were revealed for both CEW weight and pupation (S5 Fig), wherein the left-to-right gradient produced the strongest associations. ANOVA results for weight variance explained by left-to-right position were statistically significant for both control diet (R^2^ = 0.003, p = 0.0214) and test diets (R^2^ = 0.008, p < 0.0001). Logistic regression results for pupation by left-to-right position were statistically significant for both control diet (R^2^ = 0.074, p < 0.0001) and test diets (R^2^ = 0.024, p < 0.0001). To test the independence of the left-to-right positional effect from genetic entry in the case where only test diets are included (N = 9,673), two-way and full-factorial two-way ANOVAs were constructed for both the weight and pupation traits. For CEW weight, all terms were significant (p < 0.0001) in both additional models, which explained 45.9% and 48.2% of the variance, respectively. For context, entry explains 45.6% of the variance for CEW weight using this data set for one-way ANOVA. For pupation, the trends for the two additional models were very similar when only test diets are included (N = 9,673), with 37.8% and 44.2% of the variance explained, compared with 34.0% for one-way ANOVA using entry. Comparative evaluation of the gains in explanatory power across these models reveals that the interaction term showed a stronger effect than the left-to-right main effect for both the weight and pupation traits. This likely reflects the degree to which the spatial effects are dependent on test diet entries that are permissive to growth and development.

Similar trends were observed for tests involving the front-to-back gradient as the spatial term, although these were only statistically significant for the test diet subset (N = 9,673) cases for weight (R^2^ = 0.002, p < 0.0001) and pupation (R^2^ = 0.004, p < 0.0001). Notably, the full-factorial two-way ANOVA for each trait again explained the most variance, and the interaction terms accounted for more variance than the main effects for the spatial term in each model, explaining 47.7% and 37.7% for CEW weight and pupation, respectively; both of these models represent improvements over one-way ANOVAs using entry (see above). For the top-to-bottom relative positional gradient, no spatial trend was statistically detectable for either CEW weight or pupation in one-way ANOVA, however, two-way full-factorial models involving entry explained 47.0% and 37.0% for CEW weight and pupation, respectively. Both of these models represent improvements over one-way ANOVAs using entry (see above), again suggesting that the failure to grow and develop for some CEWs makes it impossible to observe ancillary effects on these processes. In summary, analyses of CEWs raised on control diet and those raised on test diets each indicate that spatial gradients within the incubation chamber account for minor, but statistically significant amounts of variance in both CEW weight gain and pupation.

### Microhabitat variability may account for spatial effects on CEWs

Even though the CEW weight and pupation effects associated with spatial gradients were minor, they offer an opportunity to consider the potential underlying causes. To this end, we leveraged microhabitat variability that was spatially recorded across the 12 bioassay runs using six temperature and relative humidity monitors placed at the bottom, middle and top of the right and left sides of the shelf system that supported the incubation trays. The six monitors were placed within a vertical plane centered between the front and the back of the shelves, meaning that no microhabitat gradients could be quantified for front-to-back within shelf, the smallest dimension of the system. Spatially consistent variation was observed among the six monitors both across the 12 bioassay runs (S6 Fig and S3 File) and averaged across the days of all experimental runs that temporally overlapped with both the preceding and succeeding bioassay runs (S7 Fig and S3 File). The sources of the microenvironmental gradients are unclear, but are presumed to arise from stabilized air-flow patterns and slight, but fixed differences in proximity to the mechanical components of the system. Shelf system placement within the incubator (see Materials and Methods) had been carefully considered at the outset of the experiments to minimize such gradients. Thus, the observation that gradients were nonetheless present emphasizes the need for both randomization and cohort stratification during incubation. The greatest microhabitat disparities were observed between opposite corners, with the top right being warmer (+1.2°C) and more humid (+7% RH) than the bottom left when averaged across bioassay runs, offering potential sources of variation that could be associated with both left-to-right and top-to-bottom gradients observed for CEW growth and development (S5 Fig).

Temperature differences are known to influence rates of CEW larval growth and development, and have been modelled extensively [33–36]. In these models, “Celsius Degree Days”, a heat unit measure of conditions that allow for growth, accumulate when temperatures are above 12.6°C and below 33.3°C. According to one CEW stage-specific phenology model [35], 202.3 Celsius degree days are required for CEW neonates to progress to pupae. The 1.2°C average difference in temperature between the warmest and coolest locations within the incubation chamber results in a difference of 13.2 Celsius Degree Days over the course of an 11 day bioassay run.

Similar to temperature, variation in relative humidity is known to affect larval development rate [36]. Our results also suggest that humidity gradients within the environmental chamber impact CEW pupation; specifically, two separate observations indicate that higher pupation rates are associated with lower relative humidity. First, pupation was highest in bioassay runs during seasons 3 & 4, which experienced conditions of lower relative humidity (Figs 5 and S6). Second, across all bioassay runs, the increase in pupation along the left-to-right gradient (S5 Fig) is congruent with a decrease in relative humidity between the left and right sides of the incubation space (S6 and S7 Figs). Interestingly, Harrell et al. [36] concluded that 70% relative humidity led to both earlier and higher levels of pupation than did 50% relative humidity, which would seemingly contradict our findings. However, the dynamic ranges of the two experiments were very different, allowing our results to augment, rather than contradict the previous findings. Because nothing close to 50% relative humidity was tested in our experiments (S6 and S7 Figs), we hypothesize that the relationship between relative humidity and pupation is non-linear, with pupation facilitated by relative humidity levels near 60%. Precise measurement of this hypothetical optimum would best be performed using conditions that can independently evaluate effects of temperature and relative humidity, such as those imposed by Harrell et al. [36].

### Summary evaluation of the quantitative CEW bioassay

The development and application of this scaled-up bioassay to evaluate maize silk resistance to corn earworm herbivory was highly successful in reliably distinguishing among quantitative resistance levels present across experimental checks and progeny families derived from Peruvian landrace Piura. To assure the possibility for detailed quantitation of resistance levels, increases in both scale and scope were undertaken. A principle driver of the increase in scale results from the choice to use a cohort of 24 CEWs per test diet, which ensures adequate biological averaging across inherently non-uniform insects. Indeed, we have shown that this level of biological replication was successful and expect that it should be used as a starting point for future CEW bioassays. In considering whether or not 24 CEWs per test diet is excessive, the potential loss of efficacy (*i*.*e*., failure to differentiate quantitative variability) must be weighed against the potential gains in efficiency, which include a reduction in field work required to produce the silks and an increase in throughput of diets evaluated per bioassay run. Because the optimal cohort size will inherently depend upon the degree of variability produced by the test diets, we suggest that optimization of cohort size should be undertaken only for cases when the bioassay is used repeatedly within the same experimental framework.

An additional driver of the increase in scale results from the choice to evaluate 1,024 CEWs per assay run. This capacity increase allows 40 test diets plus 64 CEWs raised on control diet to be evaluated within a single bioassay run, which reduces negative impacts associated with seasonal variability in the incubation environment and variability across CEW egg batches. To enable these required increases in throughput, we designed and vetted an iterative schedule for an 11-day bioassay run that commences once per week and requires only 40 hours of technical effort restricted to week days. The four day overlap between consecutive bioassay runs requires simultaneous incubation of 2,048 CEWs, presenting space usage challenges that were handled by choosing a compact layout that was not restrictive to airflow. Because we could not know how successful our space usage design would be, we tracked the positional information for every CEW and also monitored spatial and temporal variation in temperature and relative humidity within the incubator. We were able to identify spatial effects on CEW growth and development, indicating that layout matters. If we had spread the bioassay trays throughout the 3m x 3m x 2.6m incubator with no regard for proximity to lights, humidification source, or free-standing versus attached shelves, the spatial impacts would likely have been much greater, making it more difficult to attribute differences in CEW weight gain to the genetic source of maize silks. Instead, the variation in CEW growth and development attributable to spatial factors was not strong enough to merit inclusion in statistical models to explain the observed biological variation among test diets across the four Piura 208-derived populations. This technical success is likely due in part to the subdivision and spatial stratification of each cohort of 24 CEWs, which was intended to restrict the influence of spatial variation in the incubation environment. Taken together, these findings suggest that so long as replication, randomization and stratification are employed, positional tracking and microhabitat monitoring are likely superfluous efforts for incubators with only modest levels of spatial and temporal variation in temperature and relative humidity.

There are several other factors tracked in these experiments that are likely not worth the effort. For example, we recorded who performed CEW infestation and weighing and found that both processes can be affected by investigator consistency. However, cohort sub-division protects against such introduced variation, which could even be alleviated altogether if the work were done by a single investigator, which is our recommendation. Similarly, we tracked the batch and order within batch for the preparation of every test diet and found no effects, suggesting that the one-day equilibration period prior to infestation is sufficient to guard against these hypothetical sources of variation.

In summary, most of the experimental design features utilized in this work served a clear purpose and should be retained in future implementations of this quantitative bioassay. Our two-pronged approach of “monitor *and* alleviate” repeatedly showed that although the sources of experimental noise were real, they could be quashed by good experimental design to achieve a high signal-to-noise ratio.

## Future directions

The long-term goal of this work is to elucidate the genetic and biochemical mechanisms underpinning the CEW resistance discovered in silk tissue of Piura 208, a Peruvian landrace of maize (PI 503849). In this work, the quantitative CEW bioassay identified entry 100, a (GT119 x 91001)F_1:2_ family, as least permissive of CEW weight gain, the principle measure of resistance (Fig 3). We have used the remnant progeny of entry 100 to generate >500 advanced intercross doubled haploid lines (DHLs), which will facilitate detailed quantitative genetic and biochemical analyses. Several specialized metabolites have been implicated in resistance to lepidopteran herbivory of maize, belonging to the broad biochemical classes of flavonoids [15,17,19], benzoxazinoids [37,38], terpenes [39,40], oxylipins [41,42], and cuticular surface lipids [27–29]. Identification of the most resistant and most susceptible DHLs will be useful for metabolomic and entomological investigations to connect mechanism and function with causal genetic factors. When using only tens of test diets, the CEW bioassay can be augmented by quantifying differential effects on the timing of adult emergence, which lengthens generation time and creates mate-finding challenges [43,44]. Further extension could allow measuring effects on mating success and female fecundity, both of which can reduce damage even in cases where larvae are able to persist into adulthood. Lastly, Piura 208 has shown resistance to herbivory by larvae from a range of lepidopteran pests [3,21,22]. Thus, nearly isogenic contrasts among particular DHL pairs will be useful for testing efficacy against multiple pests for the individual mechanisms identified. Together, these efforts will enhance deployment of natural alleles for insect resistance that could improve food security and/or reduce grower dependence on insecticides.

## Supporting information

S1 Fig Pedigree showing derivation of the 91001 and 91007 analysis families. Breeding for this study began with two generations of selfing plants from accession 107-8-7, followed by identification of two BC_2_S_2_ families with high CEW resistance and low levels of maysins. This was accomplished using the same protocols applied in the creation of accession 107-8-7 [3]. Arrows originating below an “X” point to progeny of the depicted cross-pollination, whereas all other straight arrows denote self-pollinations and are marked by a “circled X” symbol. For both 91001 and 91007, BC_1:2_ families were derived from sets of F_1_ plants that did not overlap with those used to derive F_1:2_ families.
(TIF)

S2 Fig Percentage of CEWs that died during incubation varied across bioassay runs. Data are plotted by bioassay run, where runs 1–3 had entries of (GT119 x 91007)F_1:2_, runs 4–6 had entries of (GT119 x 91007)BC_1:2_, runs 7–9 had entries of (GT119 x 91001)BC_1:2_, and runs 10–12 had entries of (GT119 x 91001)F_1:2_. Overall, the percentage of CEWs that died during incubation was low (2.4%), but was much higher for bioassay run #9 on both control and silk-based diets, suggesting that technical factors specific to this run impacted CEW mortality.
(TIF)

S3 Fig Success of infestations with CEW neonates can vary by investigator. Mean CEW weights are plotted by individual performing infestation during each bioassay run, with percentage ‘dead at infestation’ indicated by bar color. The strongest differences in individual performance occurred during bioassay run #8. Note that “Individual 3” only infested on one date and that the number of insects affected was small (see S2 File). No data were excluded from the analyses, because the differences were minor and stratification in the experimental design minimized the impact of such technical issues in the overall evaluations.
(TIF)

S4 Fig CEW weights vary perceptibly across the investigators who handled and weighed the insects. For each bioassay season, mean CEW weights are plotted according to the individual who obtained the CEW weights. Individuals 1, 2, and 4 recorded the majority of CEW weights, with size of error bars faithfully representing the relative number of CEWs weighed by each individual during a season (S2 File).
(TIF)

S5 Fig CEW weight and percentage pupation show small levels of variation that are typically not well-explained by simple spatial gradients. In panels A, B and C, data are reported separately for control diet (N = 1641) and test diets (N = 9673), with each bar representing an average of incubation cells occupying that coordinate-positional location across all 12 runs of the bioassay. (A) Relative vertical position ordered from top-to-bottom; (B) Relative front-to-back position on shelves; (C) Relative left-to-right position on shelves using 14 bars to summarize 28 cell positions laterally arrayed across 7 trays.
(TIF)

S6 Fig Microclimates vary by location within the incubation chamber with stable trends across CEW bioassay runs. For six monitor locations, temperature and relative humidity means ± SE are reported from measurements taken every 15 minutes for each 11-day bioassay run. Minor seasonal variation is also discernable across the three sets of consecutive runs: 1–6, 7–9, and 10–12 (see Materials and Methods).
(TIF)

S7 Fig Microclimate varies by position within the incubation chamber with stable trends across the 11-day bioassay incubation period. For six monitor positions, mean temperature and mean relative humidity are plotted using data from bioassay runs 2–5, 8, and 11, none of which began or ended a consecutive set of bioassay runs (see Materials and Methods). Gray shading indicates the time period for these 6 bioassay runs when 32, rather than 64 bioassay trays were present in the incubation chamber. No statistically significant fluctuations were found to be associated with the efflux or influx of bioassay trays, indicating that the mechanical equipment regulating temperature and relative humidity is adequate for managing these abrupt two-fold changes in tray number.
(TIF)

S1 File Plot-level data for flowering time, mean CEW weight, % of CEWs that pupated by 11 days, and % of CEWs that died during incubation. The table contains 439 data rows instead of the planned 468 because 29 experimental entry plots did not produce sufficient silks for processing into test diets. Seven of the F_1:2_ entries were a complete loss, accounting for 21 failed samples. The remaining eight failed samples belonged to three F_1:2_ and four BC_1:2_ entries for which either one or two samples could not be collected. Thus, only 1.9% (8/411) of samples is missing for the analysis of the 137 entries that are represented by at least one sample, even though 6.2% (29/468) of the planned number of samples were unavailable. A key to the 12 column headings is provided.
(XLSX)

S2 File Raw data and metadata for the sampling, preparation, incubation and evaluation phases of the CEW bioassay. The table contains 12,288 data rows because it is based on the full capacity of the 12 bioassay runs. Across the 12 runs of the bioassay, 11,314 CEWs were weighed, including 266 CEWs that died during incubation but after measurable weight gain. Nonetheless, this is 974 fewer than the full capacity of 12,288 across all bioassay runs. No diet was present for 508 of the cells, mainly due to limited mass of silks for some of the test diets. In such cases, the available diet was insufficient to fill all 24 assigned cells, and was instead evenly distributed to cells across the three tray quadrants; in these cases, control diet was not used to fill the empty cells. The remaining 466 missing evaluations were due to CEWs that died prior to measurable weight gain. A key to the 26 column headings is provided.
(XLSX)

S3 File Raw temperature and relative humidity data. Sensors were placed at all 4 corners of the incubation area as well as 2 sensors placed along the horizontal boundaries at mid-height. Readings were collected every 15 or 30 minutes throughout the bioassay runs. Note that grey shading is used to indicate relative humidity data that are excluded from S6 and S7 Figs. The humidification system temporarily malfunctioned for 30 hours, causing decreased relative humidity during an overlapping incubation period for bioassay runs 3 & 4. In addition, there were instances when the monitors did not record environmental conditions; for these cases, data rows are entirely absent from the data set.
(XLSX)

S1 Table ANOVAs for cohort level CEW weight by CEW mortality. (DOCX)

S2 Table ANOVAs for CEW mortality by genotypic entry. (DOCX)
