# Supplementary figures and images for: Development and application of a quantitative bioassay to evaluate maize silk resistance to corn earworm herbivory among progenies derived from Peruvian landrace Piura
Source: PLoS One. 2019 Apr 16;14(4):e0215414. doi: 10.1371/journal.pone.0215414 (PMC6467408; doi:10.1371/journal.pone.0215414)

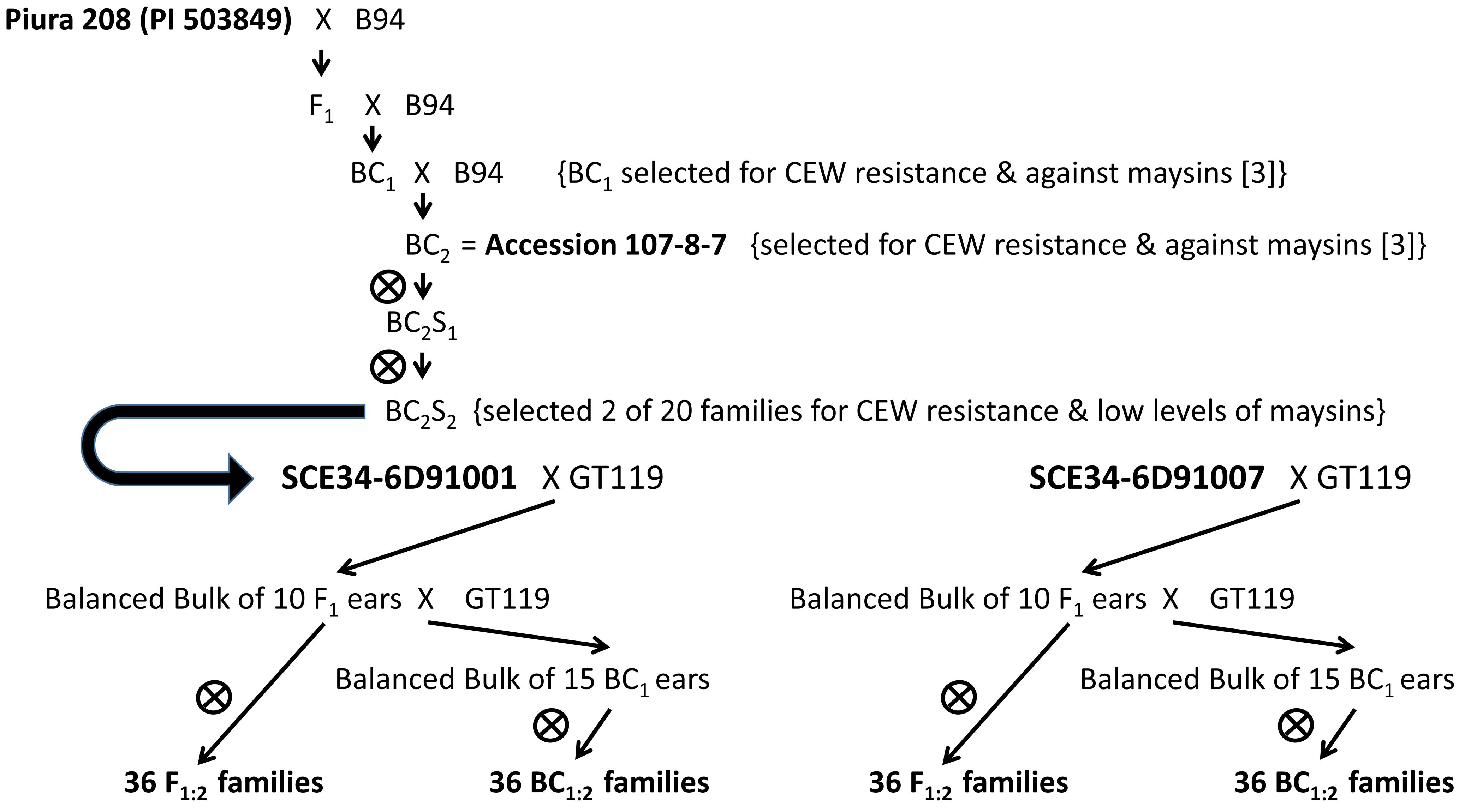

Supplement: S1 Fig — Breeding for this study began with two generations of selfing plants from accession 107-8-7, followed by identification of two BC2S2 families with high CEW resistance and low levels of maysins. This was accomplished using the same protocols applied in the creation of accession 107-8-7 [3]. Arrows originating below an “X” point to progeny of the depicted cross-pollination, whereas all other straight arrows denote self-pollinations and are marked by a “circled X” symbol. For both 91001 and 91007, BC1:2 families were derived from sets of F1 plants that did not overlap with those used to derive F1:2 families. (TIF) [file pone.0215414.s001.tif]

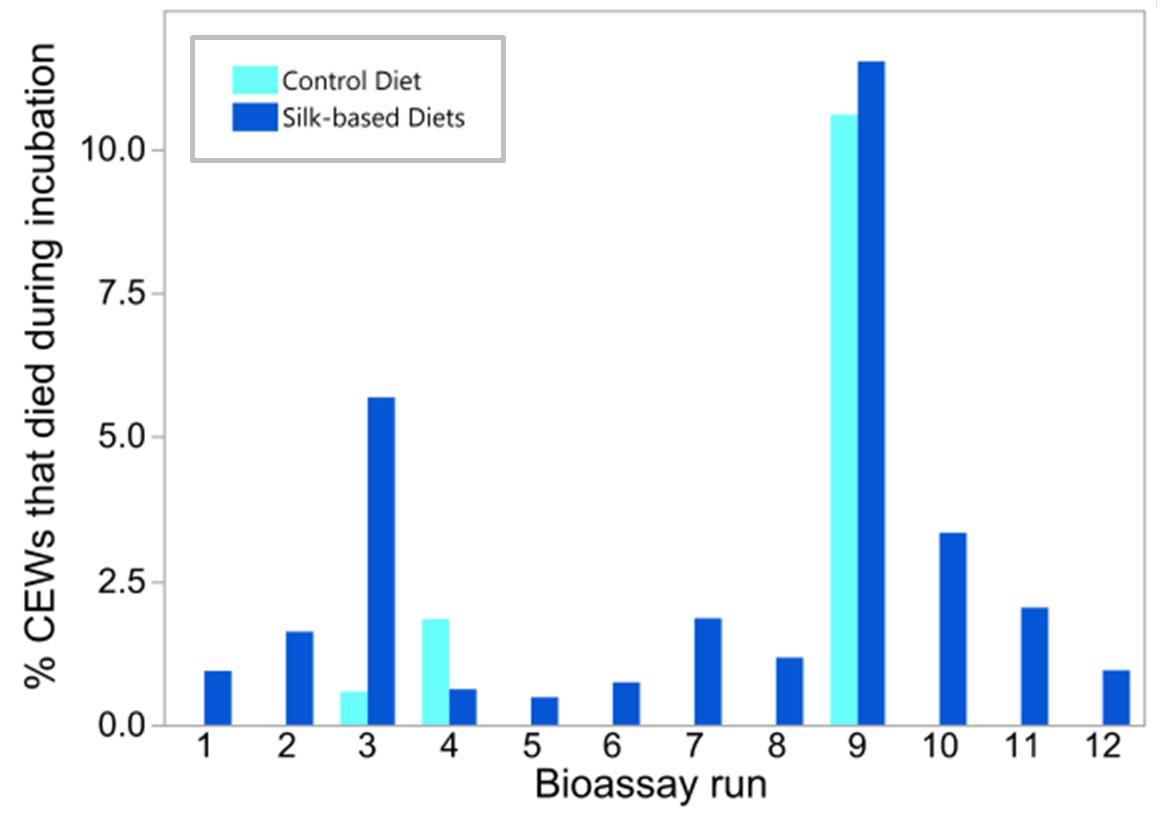

Supplement: S2 Fig — Data are plotted by bioassay run, where runs 1–3 had entries of (GT119 x 91007)F1:2, runs 4–6 had entries of (GT119 x 91007)BC1:2, runs 7–9 had entries of (GT119 x 91001)BC1:2, and runs 10–12 had entries of (GT119 x 91001)F1:2. Overall, the percentage of CEWs that died during incubation was low (2.4%), but was much higher for bioassay run #9 on both control and silk-based diets, suggesting that technical factors specific to this run impacted CEW mortality. (TIF) [file pone.0215414.s002.tif]

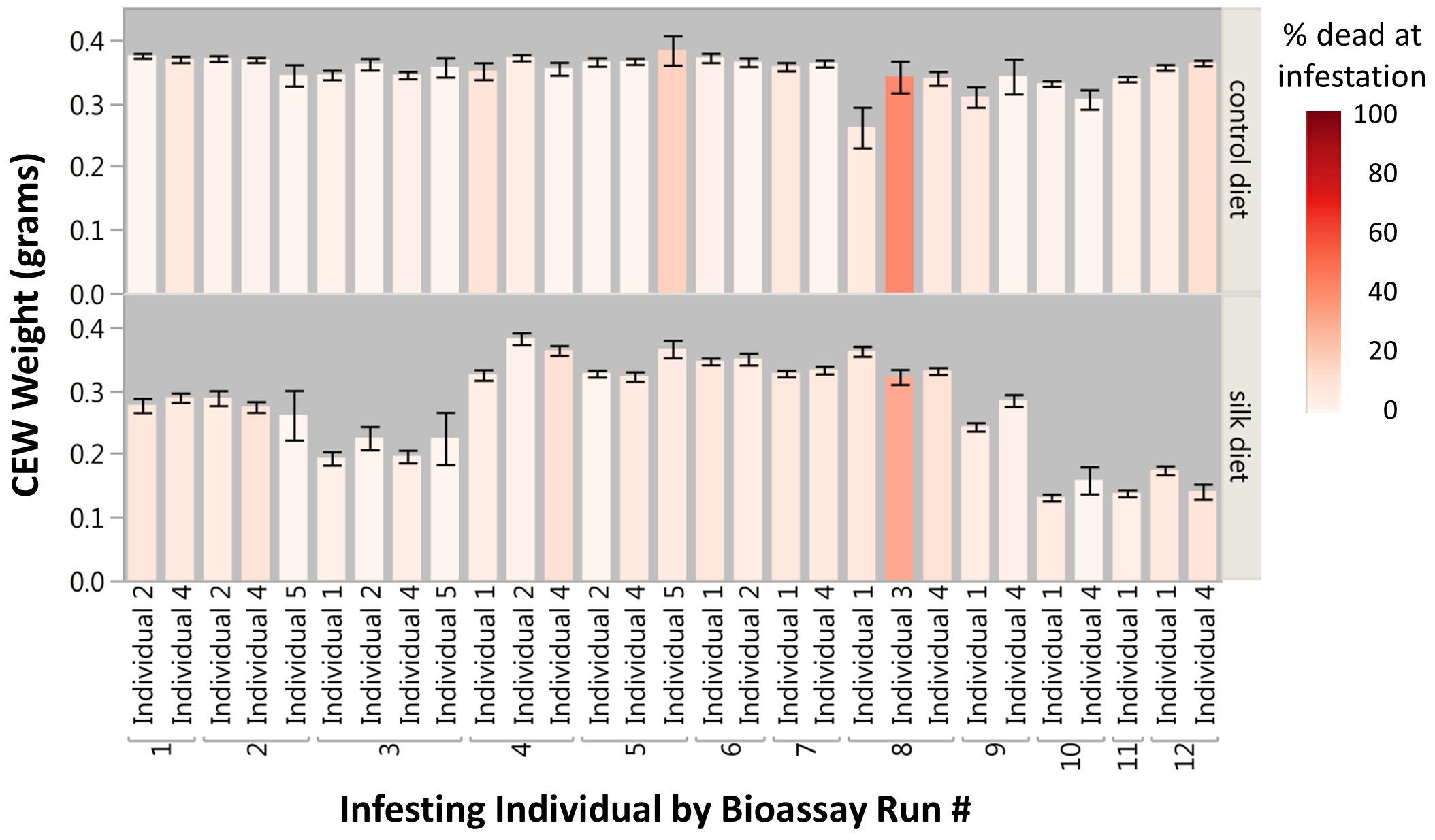

Supplement: S3 Fig — Mean CEW weights are plotted by individual performing infestation during each bioassay run, with percentage ‘dead at infestation’ indicated by bar color. The strongest differences in individual performance occurred during bioassay run #8. Note that “Individual 3” only infested on one date and that the number of insects affected was small (see S2 File). No data were excluded from the analyses, because the differences were minor and stratification in the experimental design minimized the impact of such technical issues in the overall evaluations. (TIF) [file pone.0215414.s003.tif]

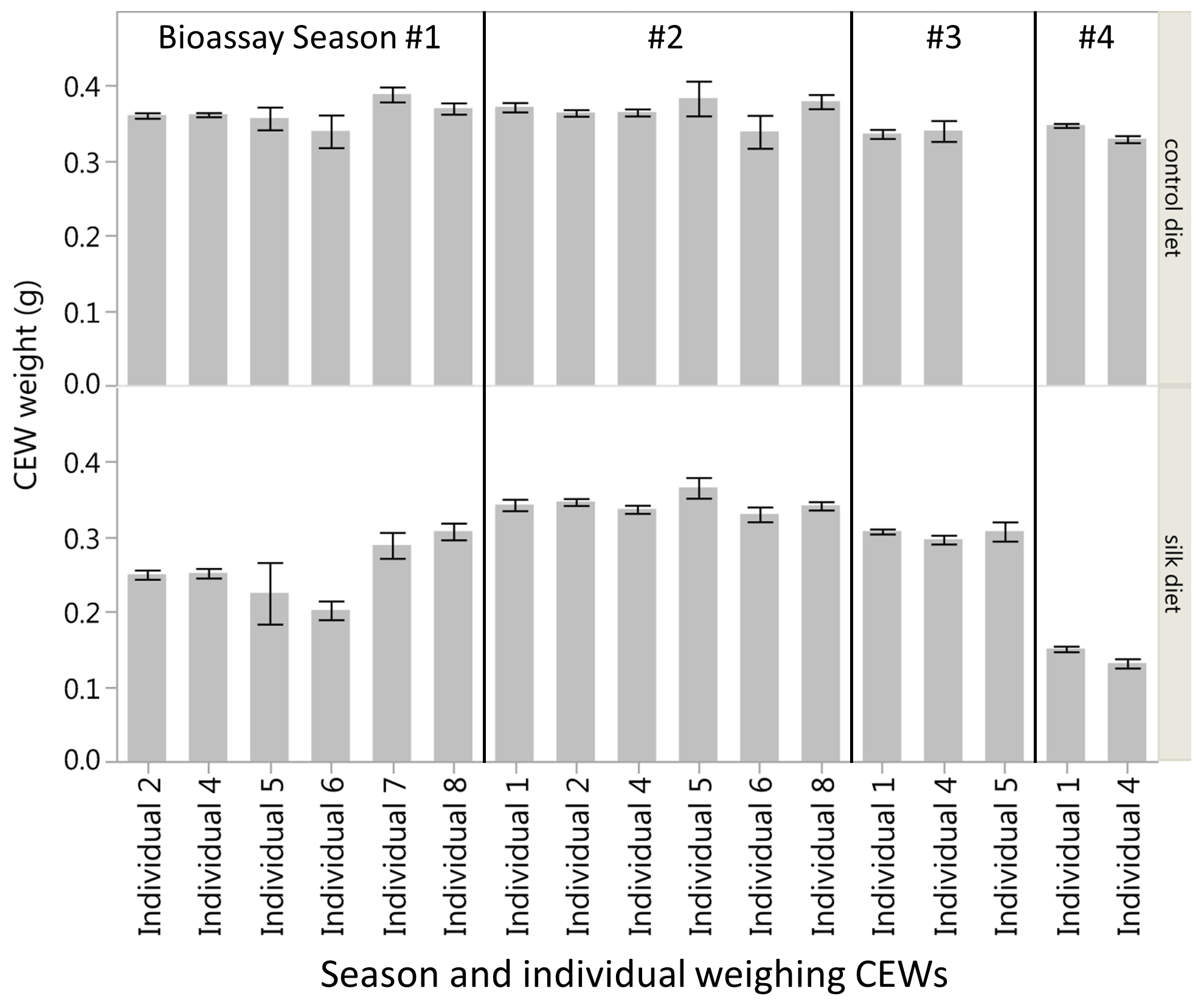

Supplement: S4 Fig — For each bioassay season, mean CEW weights are plotted according to the individual who obtained the CEW weights. Individuals 1, 2, and 4 recorded the majority of CEW weights, with size of error bars faithfully representing the relative number of CEWs weighed by each individual during a season (S2 File). (TIF) [file pone.0215414.s004.tif]

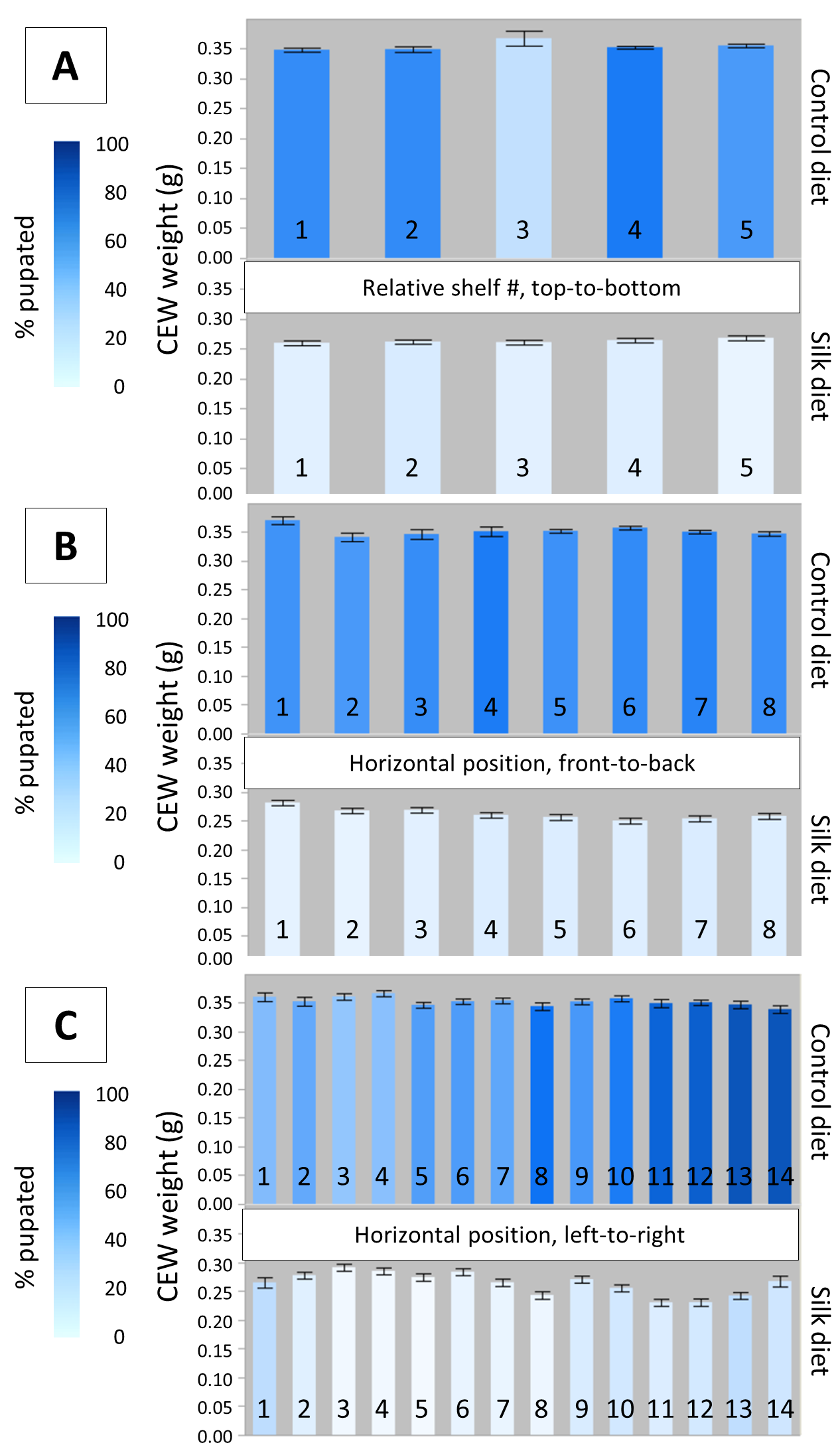

Supplement: S5 Fig — In panels A, B and C, data are reported separately for control diet (N = 1641) and test diets (N = 9673), with each bar representing an average of incubation cells occupying that coordinate-positional location across all 12 runs of the bioassay. (A) Relative vertical position ordered from top-to-bottom; (B) Relative front-to-back position on shelves; (C) Relative left-to-right position on shelves using 14 bars to summarize 28 cell positions laterally arrayed across 7 trays. (TIF) [file pone.0215414.s005.tif]

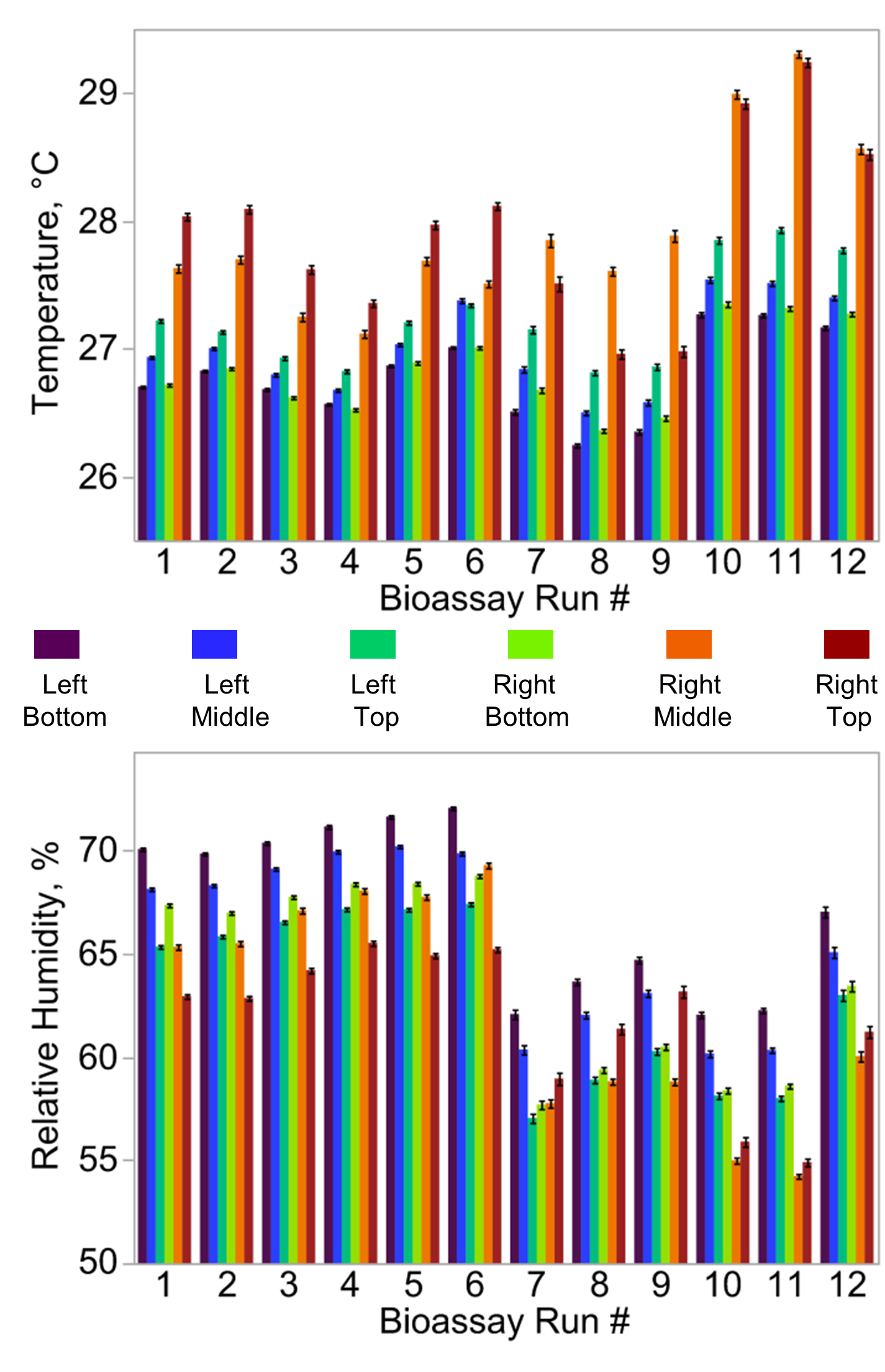

Supplement: S6 Fig — For six monitor locations, temperature and relative humidity means ± SE are reported from measurements taken every 15 minutes for each 11-day bioassay run. Minor seasonal variation is also discernable across the three sets of consecutive runs: 1–6, 7–9, and 10–12 (see Materials and Methods). (TIF) [file pone.0215414.s006.tif]

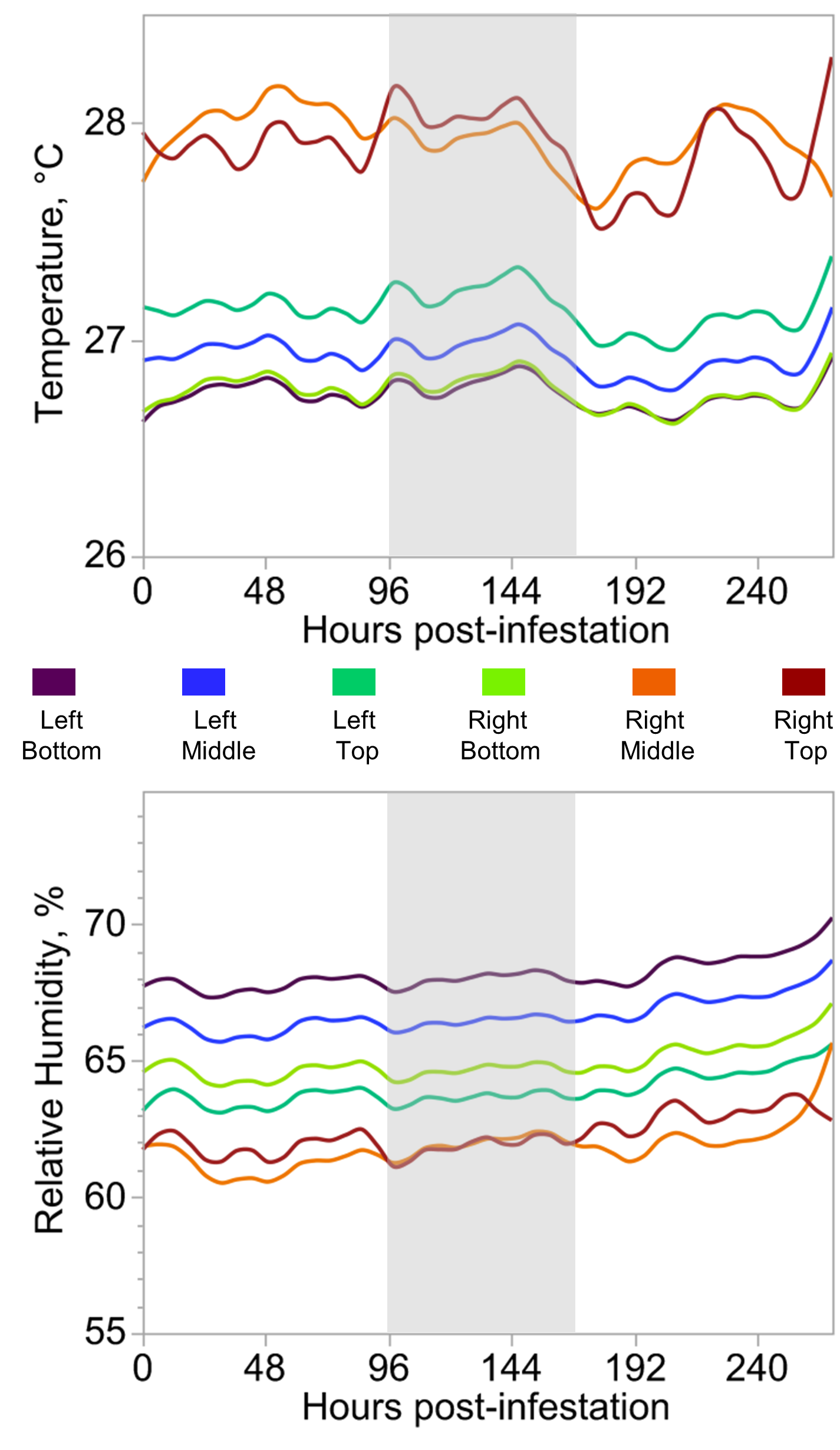

Supplement: S7 Fig — For six monitor positions, mean temperature and mean relative humidity are plotted using data from bioassay runs 2–5, 8, and 11, none of which began or ended a consecutive set of bioassay runs (see Materials and Methods). Gray shading indicates the time period for these 6 bioassay runs when 32, rather than 64 bioassay trays were present in the incubation chamber. No statistically significant fluctuations were found to be associated with the efflux or influx of bioassay trays, indicating that the mechanical equipment regulating temperature and relative humidity is adequate for managing these abrupt two-fold changes in tray number. (TIF) [file pone.0215414.s007.tif]
