# Supplementary material for: Development and application of a quantitative bioassay to evaluate maize silk resistance to corn earworm herbivory among progenies derived from Peruvian landrace Piura
Source: PLoS One. 2019 Apr 16;14(4):e0215414. doi: 10.1371/journal.pone.0215414 (PMC6467408; doi:10.1371/journal.pone.0215414)
Supplement: S1 Table — (DOCX) [file pone.0215414.s011.docx]

**S1 Table. ANOVAs for cohort level CEW weight by CEW mortality**

| **Test Group** | **N_a_** | **R^2^** | **DF_b_** | **F ratio** | **Prob >F** |
| --- | --- | --- | --- | --- | --- |
| **(GT119 x 91007)BC_1:2_** | 106 | 0.02 | 1 | 2.02 | 0.1586 |
| **(GT119 x 91001)BC_1:2_** | 69_c_ | 0.01 | 1 | 0.81 | 0.3701 |
| **(GT119 x 91007)F_1:2_** | 96 | 0.18 | 1 | 20.78 | <0.0001 |
| **(GT119 x 91001)F_1:2_** | 96 | 0.08 | 1 | 8.50 | 0.0044 |
| **Experimental check genotypes** | 33 | 0.08 | 1 | 2.85 | 0.1016 |

_a_ Sample sizes report the number of test diets assayed in each ANOVA.

_b_ Degrees of freedom are based on each bivariate fit of mean 11-day CEW weight for the living members of the cohort by the proportion of CEWs in the cohort that died after growing.

_c_ Data collected in bioassay run #9 were excluded due to exceedingly high mortality rates, even among CEWs raised on control diet.
