# Supplementary material for: Development and application of a quantitative bioassay to evaluate maize silk resistance to corn earworm herbivory among progenies derived from Peruvian landrace Piura
Source: PLoS One. 2019 Apr 16;14(4):e0215414. doi: 10.1371/journal.pone.0215414 (PMC6467408; doi:10.1371/journal.pone.0215414)
Supplement: S2 Table — (DOCX) [file pone.0215414.s012.docx]

**S2 Table. ANOVAs for CEW mortality by genotypic entry**

| **Test Group** | **N_a_** | **R^2^** | **DF_b_** | **F ratio** | **Prob >F** |
| --- | --- | --- | --- | --- | --- |
| **(GT119 x 91007)BC_1:2_** | 106 | 0.28 | 35 | 0.78 | 0.7929 |
| **(GT119 x 91001)BC_1:2_** | 69_c_ | 0.41 | 35 | 0.64 | 0.8997 |
| **(GT119 x 91007)F_1:2_** | 96 | 0.24 | 31 | 0.64 | 0.9143 |
| **(GT119 x 91001)F_1:2_** | 96 | 0.33 | 32 | 0.97 | 0.5206 |
| **Experimental check genotypes** | 33 | 0.26 | 5 | 1.86 | 0.1350 |

_a_ Sample sizes report the number of test diets assayed in each ANOVA.

_b_ Degrees of freedom are based on number of corn genotype entries in each ANOVA.

_c_ Data collected in bioassay run #9 were excluded due to exceedingly high mortality rates, even among CEWs raised on control diet.
